# Integrative Analysis of Immune-Related Genes in the Tumor Microenvironment of Renal Clear Cell Carcinoma and Renal Papillary Cell Carcinoma

**DOI:** 10.3389/fmolb.2021.760031

**Published:** 2021-11-23

**Authors:** Bin Zheng, Fang Xie, Fajuan Cheng, Jianwei Wang, Zhongshun Yao, Wei He, Zhihong Niu

**Affiliations:** ^1^ Cheeloo College of Medicine, Shandong University, Jinan, China; ^2^ Department of Urology, Shandong Provincial Hospital Affiliated to Shandong First Medical University, Jinan, China; ^3^ Department of Urology, Shandong Provincial Hospital Affiliated to Shandong University, Jinan, China; ^4^ Department of Urology, Weihai Municipal Hospital, Weihai, China; ^5^ Department of Nephrology, Shandong Provincial Hospital Affiliated to Shandong University, Jinan, China; ^6^ Department of Nephrology, Shandong Provincial Hospital, Cheeloo College of Medicine, Shandong University, Jinan, China; ^7^ Department of Urology, Shandong Provincial ENT Hospital Affiliated to Shandong University, Jinan, China

**Keywords:** clear cell renal carcinoma, papillary renal cell carcinoma, prognosis, tumor microenvironment, immunotherapy

## Abstract

Kidney cancer encompasses a range of primary cancers, such as clear cell renal cell carcinoma (ccRCC) and papillary renal cell carcinoma (pRCC). Our knowledge about the tumor microenvironment (TME) of kidney cancer is still limited. Therefore, we comprehensively assessed the TME of kidney cancers (including ccRCC and pRCC) using the ESTIAMTE, and CIBERSORT algorithms, and conducted distinct functional and correlation analyses with data from The Cancer Genome Atlas (TCGA), International Cancer Genome Consortium (ICGC), Gene Expression Omnibus (GEO), Connectivity map and CellMiner database. Next, we identified two immune-related hub genes, *IGLL5* and *IL2RA*, which play essential roles in the TME as well as on survival in ccRCC and pRCC. Furthermore, ccRCC and pRCC samples from our medical center were collected to verify the clinical application value of these two immune-related genes. A specific enrichment analysis of immune cells related to *IGLL5* and *IL2RA* was also conducted in two types of renal cell cancer. Based on selected genes, we predicted the drug response and uncovered novel drug candidate for RCC treatment. Considering the unfavorable outcomes of kidney cancer and emerging interest in TME-targeted treatments, our results may offer insights into immune-related molecular mechanisms and possible targets to control the kidney cancer.

## Introduction

Kidney or renal cancer is the sixth most common malignant cancer in men and the ninth most common in women. In 2020, it was estimated that 76,080 patients would be diagnosed with kidney cancer and 13,780 patients would die from the disease in the United States (Siegel et al., 2021). Renal cell carcinoma (RCC) is the most common form and accounts for 85% of all histological types, with ccRCC comprising 70–80% and pRCC 15–20% of all RCC (Linehan et al., 2016; Barata and Rini, 2017; Vuong et al., 2019). Although pRCC has a better prognosis than ccRCC, the overall prognosis for ccRCC and pRCC remains limited (Steffens et al., 2012). Therefore, effective therapy and accurate biomarkers need to be determined.

TME, as an integral part of tumors, is a cellular environment consisting of tumor cells and other non-malignant cells, including surrounding immune cells, lymphocytes, fibroblasts, stromal cells, and blood vessels (Wu and Dai, 2017). TME acts as a complex ecosystem, which could support tumor growth as well as metastasis while attenuating immunosurveillance. A wealth of new information has emerged which reveals how the functionality of TME determines its integral and indispensable role in various cancers (Hanahan and Coussens, 2012; Pitt et al., 2016). Within the TME, various types of immune and non-immune cell are found. Some nontumor cells, such as stromal cells, fibroblasts, may accelerate cancer cell proliferation and stimulate cancer progression (Hanahan and Coussens, 2012). However, although several immune cells, such as T cells as well as B cells also exist in TME, the exact contribution of these immune cells to the prognosis of patients is not clear (Grivennikov et al., 2010). With a variety of factors that diverse cells secrete, these lead to a chronic inflammatory, immunosuppressive environment (Osipov et al., 2019). Within that environment, cancer cells are able to adapt and grow with less possibility of detection and eradication by immunosurveillance.

Currently, RCC is considered an immunogenic and vascularized tumor. Several studies have found that immune cells could infiltrate into the TME of RCC, but these immune cells block the effective anti-tumor responses (Díaz-Montero et al., 2020). Due to the immunosuppressed state of RCC, attention has focused on immune checkpoint inhibitors. However, a large number of patients with RCC do not benefit from these immune-based treatments, and therapies are related with drug resistance because of heterogeneous and adaptive TME (Ravaud et al., 2016). In order to eradicate tumor cells, effector immune cells must be relieved from the immunosuppressive networks that constitute the TME. Moreover, with the improved understanding of interactions between immune cells, and between cancer cells and immune cells, the optimizing of our approach to treating cancers will significantly progress (Joyce and Pollard, 2009). Therefore, it is indispensable to elucidate the cross-talk process between various immune cells and the TME.

In this study, we identified the immune-related genes in the TME of ccRCC as well as pRCC, and explored their potential mechanisms, biological functions, and predict drug response as well as potential drugs for ccRCC and pRCC. These immune-genes could serve as reliable prognostic biomarkers as well as therapeutic targets.

## Materials and Methods

### Data Collection

The gene expression data and clinical information of ccRCC (539 ccRCC and 72 normal samples) and pRCC (289 pRCC and 32 normal samples) were downloaded directly from TCGA (https://portal.gdc.cancer.gov/). The gene expression data and clinical information of 91 and 61 RCC samples were obtained from ICGC (https://dcc.icgc.org/) and GEO (including GSE40912 and GSE2748) (https://www.ncbi.nlm.nih.gov/geo/), separately. We eliminated samples in which clinical information was lost or unknown.

### Identification and Analysis of Differentially Expressed Genes

Differentially expressed genes (DEGs) were identified using the “limma” package and visualized using the “pheatmap” package. The |logFC| > 1, *p*-value < 0.05, and false discovery rate (FDR) < 0.05 were used to screen for DEGs. The correlation between immune-related genes and clinical factors was performed using the “ggplot2” and “corrplot” package.

### Functional Analysis

Protein–protein interaction (PPI) network construction was performed using the STRING online database (http://string-db.org/), and the network was visualized using Cytoscape (Szklarczyk et al., 2019). Gene Ontology (GO) and Kyoto Encyclopedia of Genes and Genomes (KEGG) analyses was conducted by “clusterProfiler” package. Gene set enrichment analysis (GSEA) was performed by the “gsva” R package. The Connectivity Map (Cmap) and CellMiner database were uesd to predict small molecular compounds and individual response to drugs.

### Tumor Microenvironment Analysis

We used ESTIMATE algorithm (Estimation of Stromal and Immune cells in Malignant Tumor tissues using Expression data) algorithm to measure stromal and immune scores in ccRCC and pRCC with data from TCGA database (https://portal.gdc.cancer.gov/) (Yoshihara et al., 2013). The prognostic-related genes were selected after performing the Univariate Cox analysis with *p* < 0.1. Tumor-infiltrating immune cells were calculated by the CIBERSORT algorithm. Venn diagrams as well as violin plots were visualized with the ‘VennDiagram’ and ‘vioplot’ packages.

### Immunohistochemistry

Ten pairs of ccRCC, pRCC and adjacent normal tissues were collected from Shandong provincial Hospital affiliated to Shandong First University from April 2021 to June 2021. The study was approved by the Ethics Committee of Shandong Provincial Hospital (Approval no. SWYX: NO. 2021-118). IHC was performed according to previously published method (Wang et al., 2020). All samples were incubated with rabbit polyclonal anti-IL2RA (ab245687) and anti-IGLL5 (NBP2-14574) antibodies overnight at 4°C and then washed. Two pathologists independently assessed the IHC slides.

### Statistical Analysis

Mann-Whitney test was utilized to measure gene expression between tumor and non-tumor tissues. The Kaplan-Meier curves with a log-rank test were adopted in the survival analysis. Statistical analysis in this study was performed with the R statistical package (R version 4.0.1).

## Results

### Correlation of ESTIMATE Scores in ccRCC and pRCC

**FIGURE 1:**
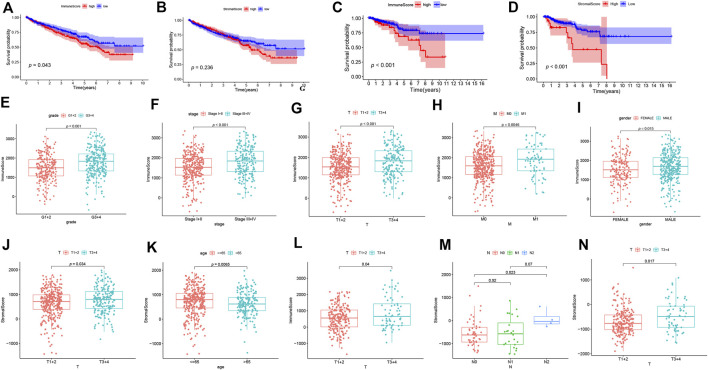
The correlation analysis between ESTIMATE scores and prognosis of patients. **(A-D)** The K-M curves for ESTIMATE scores in ccRCC **(A-B)** and pRCC **(C-D)**. **(E-N)** The correlation analysis between ESTIMATE scores and clinicopathological features in ccRCC **(E-K)** and pRCC **(L-N)**.

We first determined the association between ESTIMATE scores (including immune scores and stromal scores), prognosis as well as clinical characteristics in ccRCC and pRCC. As shown in Figures 1A,B, ccRCC patients with high immune scores had poor prognosis, but stromal scores did not have apparently association with survival. However, in pRCC patients, a decrease in overall survival was correlated with high immune scores and stromal scores (Figures 1C,D). In addition, for ccRCC, immune scores were positively associated with tumor grade, tumor stage, T stage, and M stage. Patients with advanced T stage had relatively higher stromal scores (Figures 1E–K). For pRCC, higher immune scores were associated with T stage, while patients with advanced T stage and N stage have high stromal scores (Figures 1L–N).

### Identification of Differentially Expressed Genes

**FIGURE 2:**
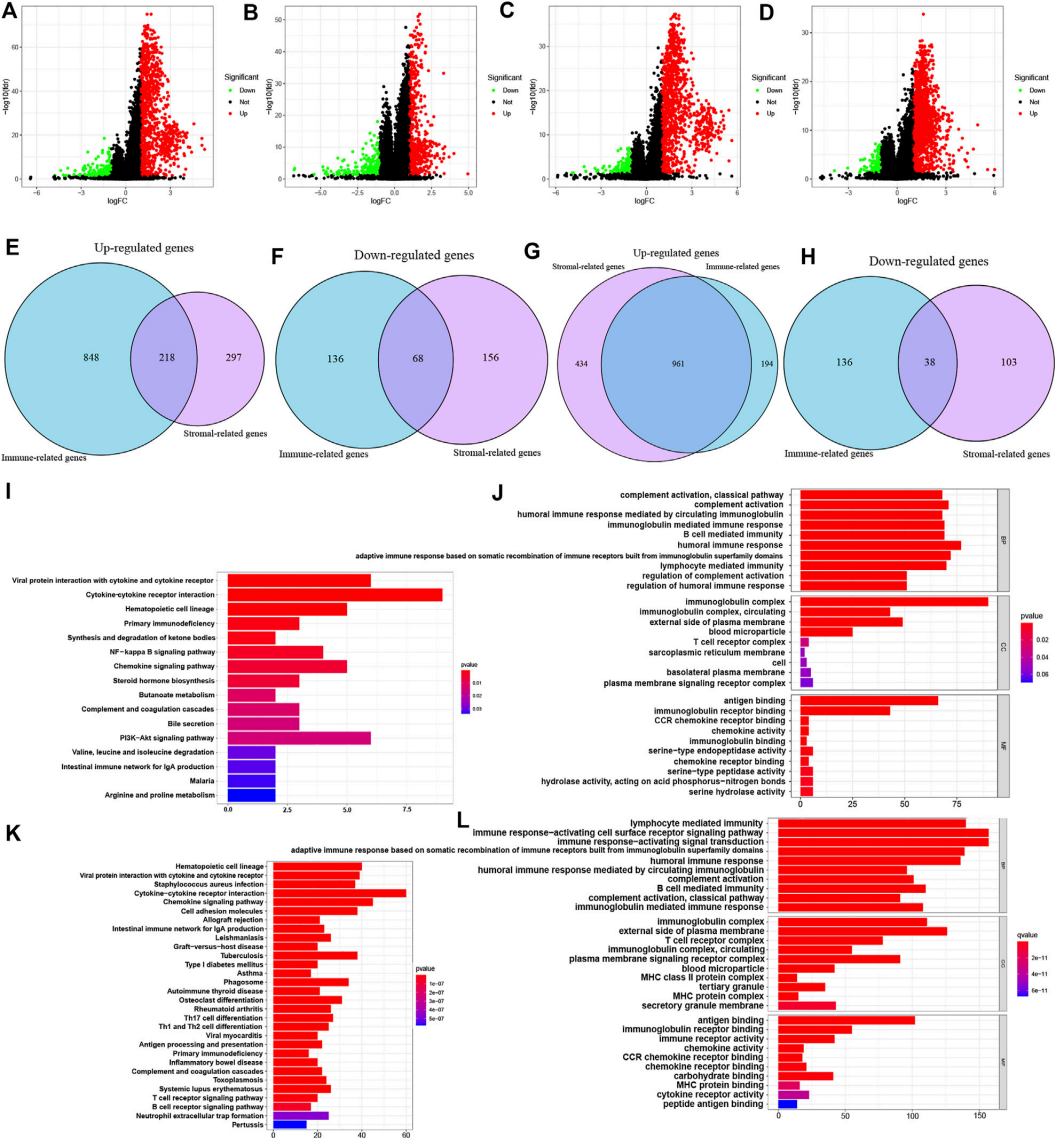
Identification of DEGs based on ESTIMATE scores and functional analysis of common DEGs. **(A-D)** Volcano plot of DEGs from the immune scores and stromal score groups in ccRCC **(A-B)** and pRCC **(C-D)**. **(E-H)** Commonly changed genes in immune scores and stromal scores groups in ccRCC **(E-F)** and pRCC **(G-H)**. **(I-L)** KEGG and GO enrichment analysis in ccRCC **(I-J)** and pRCC **(K-L)**.

Based on immune scores and stromal scores, we determined the DEGs by analyzing the expression data of all ccRCC and pRCC samples. In ccRCC, 1066 genes were upregulated and 204 genes were downregulated in samples with high immune score. Similarly, we also identified 515 genes that were highly expressed and 221 genes with low expression in ccRCC samples with high stromal score (logFC >1; *p* < 0.05; Figures 2A,B). In pRCC, 1155 genes had high expression levels and 174 genes had low expression in patients with high immune scores, and 1395 genes were upregulated as well as 141 downregulated in patients with high stromal scores (Figures 2C,D). Using Venn plot, we determined 218 genes with high expression and 68 genes with low expression in ccRCC patients with high scores (Figures 2E,F). pRCC patients with high scores presented 961 genes that were upregulated and 38 genes that were downregulated, respectively (Figures 2G,H).

On the basis of these selected DEGs, we performed GO and KEGG enrichment analyze in two types of kidney cancers. The GO analysis showed that DEGs identified in ccRCC and pRCC cases were primarily enriched in immune-related functions, such as B cell-mediated immunity and lymphocyte-mediated immunity (Figures 2J–L). We also listed the top 10 KEGG pathways for both ccRCC and pRCC. As shown in Figures 2I–K, various common immune-related signaling pathways were enriched. Furthermore, DEGs from pRCC samples were also enriched in several pathways related to T-cell receptor and B-cell receptor signaling pathways (Figure 2K).

### PPI Network and Prognostic Analysis of DEGs

**FIGURE 3:**
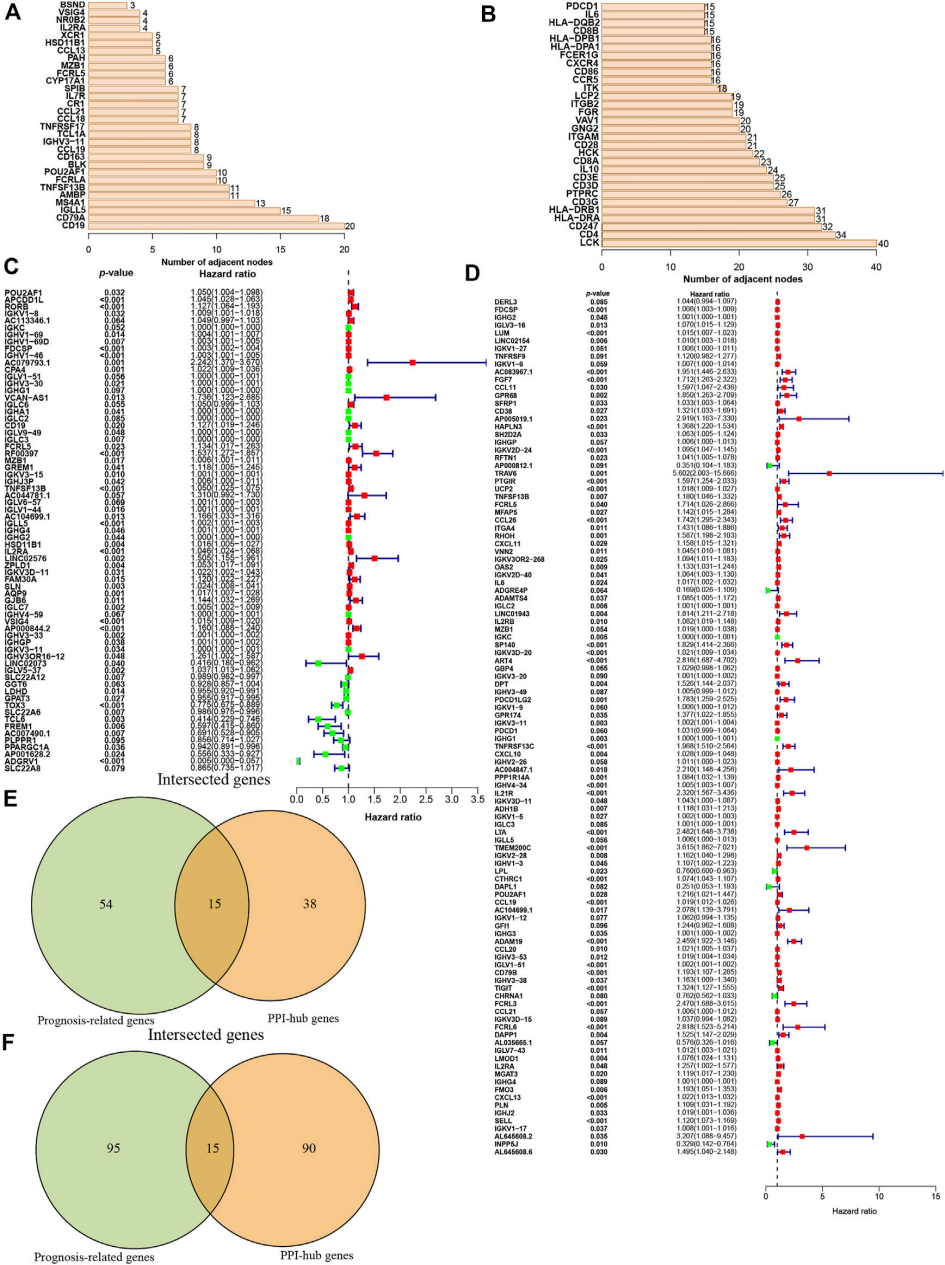
Identification of the prognostic hub DEGs. **(A-B)** Hub DEGs ranked by the number of nodes in ccRCC **(A)** and Prcc **(B)**. **(C-D)** Construction of univariate Cox analysis in ccRCC **(C)** and pRCC **(D)**. **(E-F)** Identification of prognostic hub DEGs *via* Venn diagram in ccRCC **(E)** and pRCC **(F)**.

Then these identified DEGs were used to construct the PPI networks. We ranked the DEGs genes by their nodes (Figures 3A,B) and identified hub genes *via* “Cytoscape” (Supplementary Figures S1A–D). Moreover, we further investigated prognostic genes by performing the univariate Cox analysis among all DEGs in ccRCC and pRCC, respectively. This revealed that 69 genes were related to ccRCC prognosis, and 110 genes were associated with the prognosis of pRCC patients (*p* < 0.1, Figures 3C,D). Then, we interrogated the top 15 prognosis-related hub genes in ccRCC and pRCC utilizing Venn algorithm (Figures 3E,F). Among 30 selected prognosis-related hub genes in ccRCC and pRCC, two common genes, *IGLL5* and *IL2RA*, were identified, which could potentially play essential role in TME as well as survival in ccRCC and pRCC.

### Correlation and GSEA Enrichment Analyses of *IGLL5* and *IL2RA*

**FIGURE 4:**
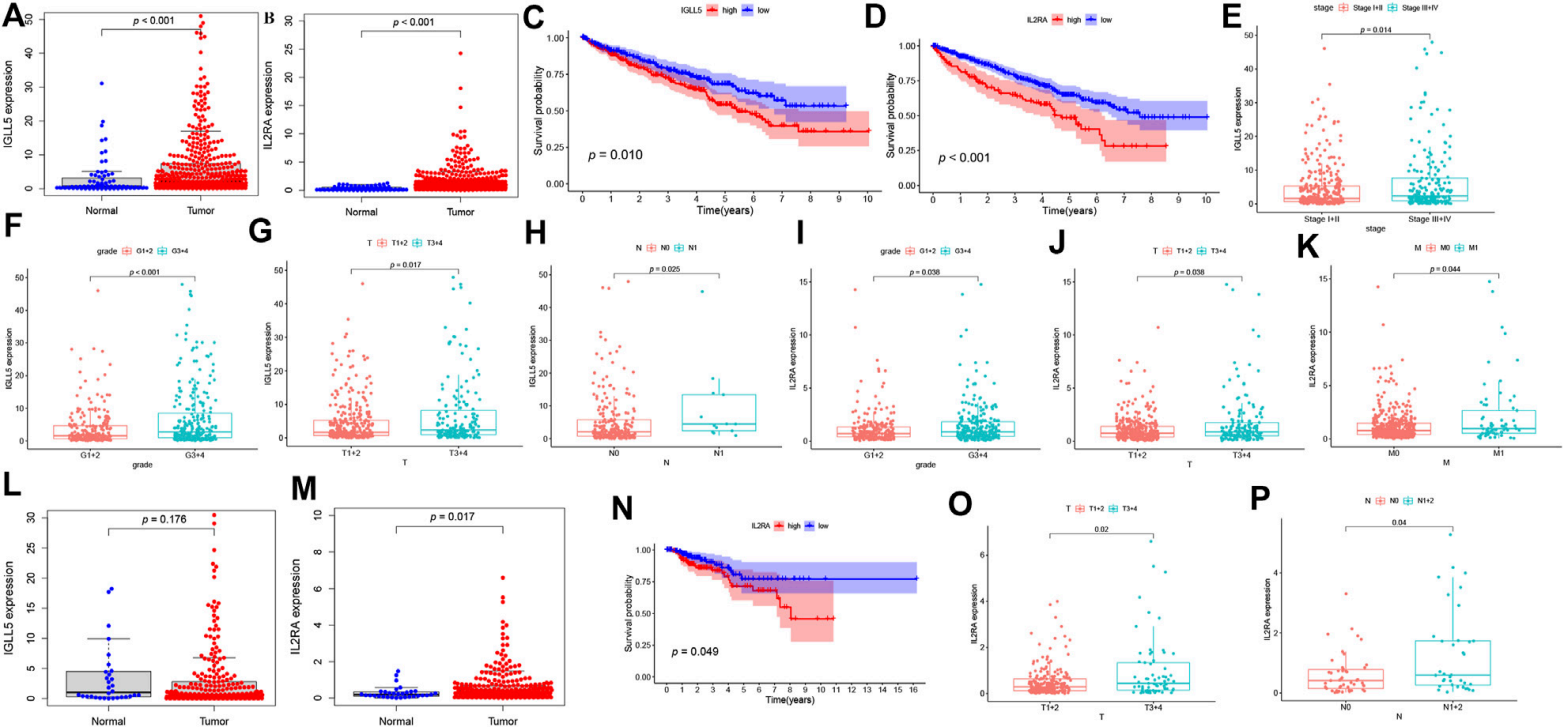
Survival analysis and the clinic correlation analysis in ccRCC and pRCC. **(A-B, L-M)** Differential expression analysis of *IGLL5* and *IL2RA* in ccRCC **(A-B)** and pRCC samples **(L-M)**. **(C-D)** Survival analysis of *IGLL5* and *IL2RA* groups in ccRCC and pRCC samples **(N)**. **(E-K)** The correlation analyses of *IGLL5* **(E-H)** and *IL2RA* **(I-K, O-P)** expression with clinicopathological parameters in ccRCC and pRCC samples.

To further assess the expression profile and clinical values of *IGLL5* and *IL2RA*, we extracted the corresponding expression data, as well as clinical information. Both *IGLL5* and *IL2RA* showed high expression in ccRCC samples (*p* < 0.05, Figures 4A,B). Furthermore, a decrease in overall survival was associated with high expression of *IGLL5* and *IL2RA* (*p* < 0.05, Figures 4C,D). *IGLL5* and *IL2RA* expression were also positively associated with the stage of ccRCC patients, including high tumor stage, tumor grade, T stage, and more lymph node metastases (*p* < 0.05, Figures 4E–K).

The same approach was used to analyze two genes in pRCC patients. The differential expression analysis confirmed that *IL2RA* expressed highly in tumor as well (*p* < 0.05, Figure 4M), but significant differences in expression of *IGLL5* between normal and tumor patients was not observed (*p* > 0.05, Figure 4M). Based on these results, we chose *IL2RA* for further analysis. In pRCC patients, poor survival was associated with high expression of *IL2RA* (*p* < 0.05, Figure 4N). Moreover, high *IL2RA* expression was associated with various clinical characteristics, including T and N stage indicative of disease progression. (*p* < 0.05, Figures 4O,P).

Next, we explored the potential regulation pathways through GSEA enrichment analysis in ccRCC and pRCC samples. Several immune-related pathways, including B cell and T cell receptor signaling pathways, chemokine signaling, and natural killer cell mediated cytotoxicity, which were closely correlated with *IGLL5* and *IL2RA* expression (NES >1, *p* < 0.05, FRD <0.25, Supplementary Figures S2A–C).

### Composition of Immune Cells and Correlation Analysis

**FIGURE 5:**
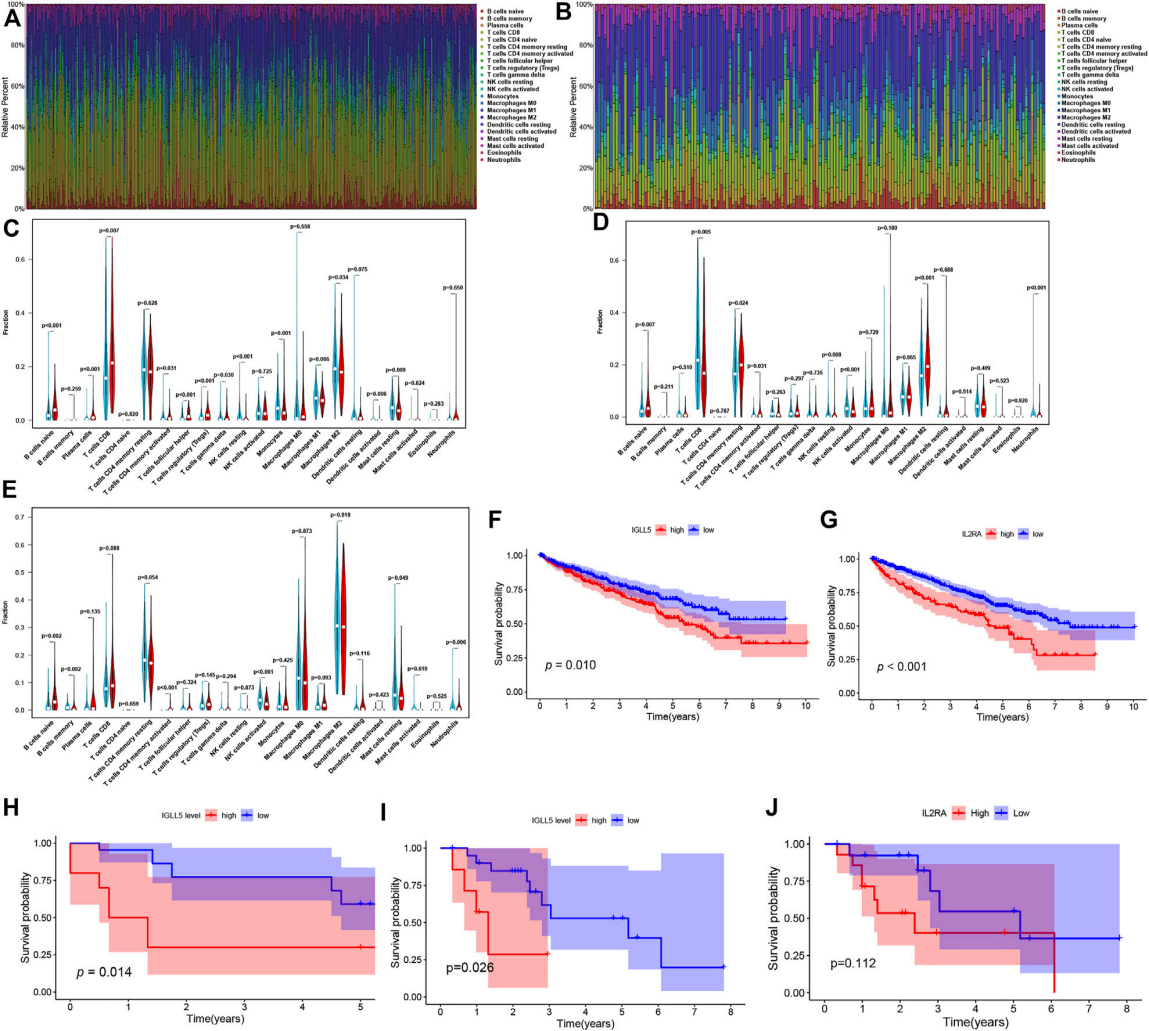
Composition of immune cells and correlation analysis. **(A,B)** landscape of the 22 Immune cell in ccRCC **(A)** and pRCC **(B)**. **(C-E)** Differences of immune cells infiltration in ccRCC **(C,D)** and pRCC **(E)**. **(F-I)** Validation of *IGLL5* and *IL2RA* in ICGC cohort **(F-G)**, GSE40912 **(H)** and GSE2748 **(I-J)**.

To further verify the essential role of major immune cells in ccRCC and pRCC, we explored immune cell infiltration and correlation between two genes and major immune cells. The profile of the principle immune cells showed that adaptive immune cells (such as native B cells, memory B cells, native and memory CD4^+^ cell, CD8^+^ T cells) accounted for a substantial part of the immune cells in ccRCC as well as pRCC (Figures 5A,B). Next, we investigated differences in the fraction of major types of immune cells according to expression levels of *IGLL5* and *IL2RA*. For ccRCC, high *IGLL5* expression was directly proportional to most adaptive immune cells (including native B cell, CD4^+^ memory T cells, CD8^+^ T cells, T follicular helper cells, and regulatory T cells) and was inversely proportion to several innate immune cells (such as NK cell, monocytes, M2 macrophages, activated dendritic cells, and mast cells) (*p* < 0.05, Figure 5C). Moreover, the difference analysis between high and low expression level of *IL2RA* in ccRCC illustrated that the high *IL2RA* expression set presented a significantly larger fraction of native B cells, CD4^+^ memory T cells, and M2 macrophages, than CD8^+^ T cells, NK cells, and neutrophils (*p* < 0.05, Figure 5D). The same approach was used to assess *IL2RA* in pRCC, which revealed that high *IL2RA* expression set had higher fraction of native B cells, neutrophils, and a lower proportion of memory B cells, activated CD4^+^ T memory cells, activated NK cells and resting mast cells (*p* < 0.05, Figure 5E).

We also detected the correlation of *IGLL5* and *IL2RA* with immune cell abundance. In ccRCC, the correlation analyses showed that *IGLL5* and most adaptive immune cells had a clear linear positive correlation and had a negative correlation with the infiltration of native immune cells (*p* < 0.05, Supplementary Figure S3). For *IL2RA*, there was a positive relationship with native B cells, activated CD4^+^ memory T cells, and M0 and M2 macrophages. However, an inverse correlation was observed between *IL2RA* and CD8^+^ T cells, T follicular helper cells, and NK cells (*p* < 0.05, Supplementary Figure S4). In pRCC, *IL2RA* showed a positive correlativity with activated CD4^+^ T memory cells, native B cells, M1 macrophages and neutrophils but had negative relationship with resting CD4^+^ T memory cells, memory B cells, and mast cells (*p* < 0.05, Supplementary Figure S5). Finally, on the basis of above analyses, we further investigated the intersected immune cells associated with both *IGLL5* and *IL2RA* in ccRCC and pRCC (Table. 1).

**TABLE 1:** The intersected immune cells related to *IGLL5* and *IL2RA* in ccRCC and pRCC.

Cancer types	KIRC				KIRP	
Gene	IGLL5		IL2RA		IL2RA	
	Immune cells	*p*-Value	Immune cells	*p*-Value	Immune cells	*p*-Value
	B cells naive	<0.01	B cells naive	0.001	B cells naive	0.001
	Plasma cells	<0.01	T cells CD8	0.0002	B cells memory	0.001
	T cells CD8	0.008	T cells CD4 memory activated	<0.01	Plasma cells	0.015
	T cells CD4 memory activated	0.005	T cells follicular helper	0.044	T cells CD4 memory resting	0.001
	T cells follicular helper	0.001	NK cells resting	0.017	T cells CD4 memory activated	<0.01
	T cells regulatory (Tregs)	<0.01	NK cells activated	<0.01	NK cells activated	<0.01
	T cells gamma delta	0.026	Macrophages M0	0.004	Macrophages M1	0.036
	NK cells resting	<0.01	Macrophages M2	<0.01	Mast cells resting	0.013
	Monocytes	0.0004	Neutrophils	<0.01	Neutrophils	0.001
	Macrophages M2	0.002				
	Dendritic cells activated	0.019				
	Mast cells resting	0.003				

### Prognostic Value of IGLL5 and IL2RA in Validation Cohort

We utilized RCC data from the ICGC and GEO database to validate the prognostic value as well. In RCC patients from the ICGC database, a decrease in overall survival was significantly related to the high expression of *IGLL5* and *IL2RA* (*p* < 0.05, Figures 5F,G). We then explored the prognostic value of *IGLL5* and *IL2RA* using data from GSE40912 (n = 32 samples) and GSE2748 (n = 28 samples) datasets. The results indicated that high expression of *IGLL5* was significantly associated with poor prognosis (*p* < 0.05, Figures 5H,I). Patients with high expression of *IL2RA* had poor survival, but the difference was not statistically significant (*p* = 0.112, Figure 5J)

**FIGURE 6:**
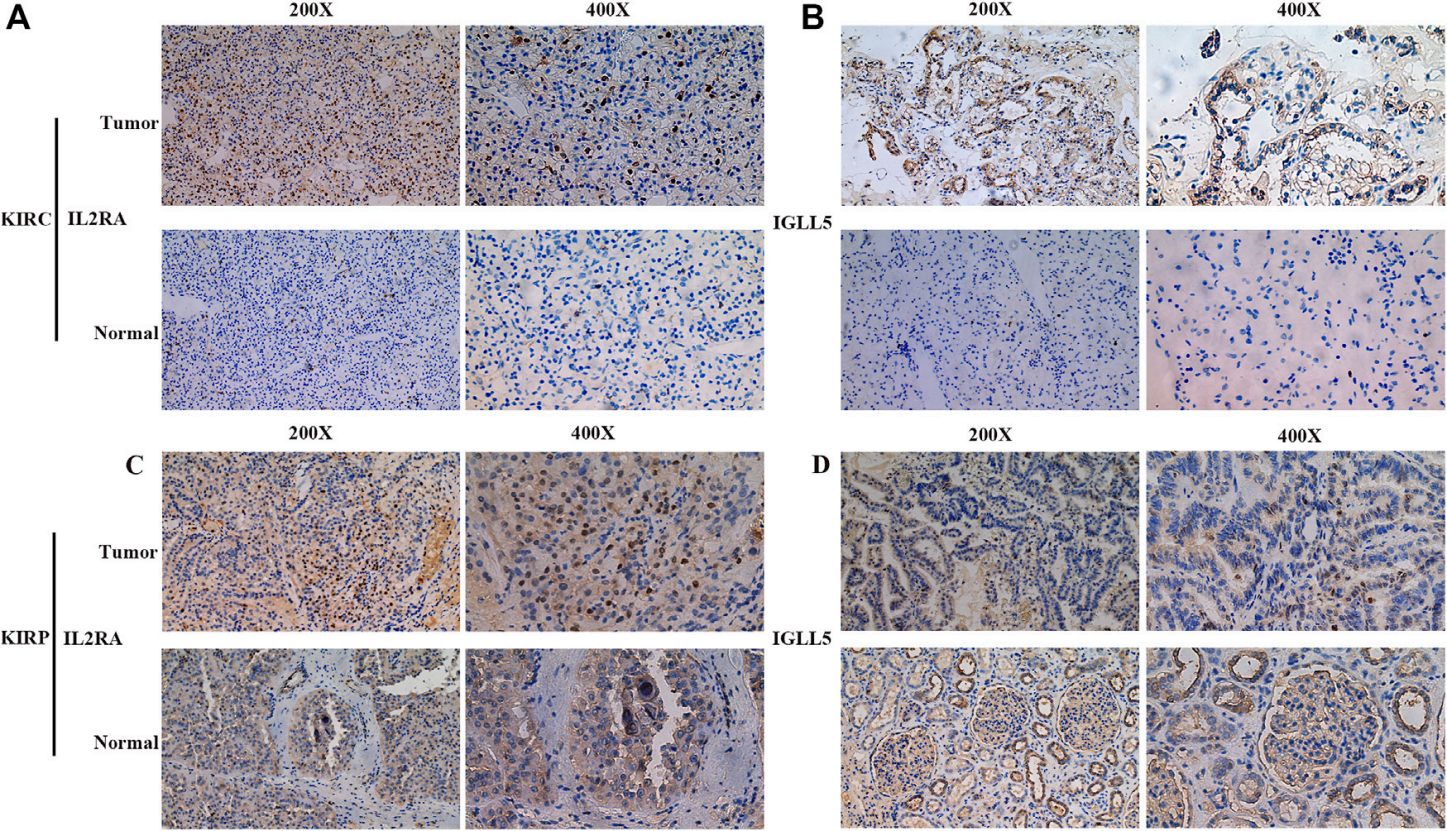
Representative IHC images of *IGLL5* and *IL2RA* in ccRCC and pRCC samples. **(A,B)** IHC patterns for *IL2RA* (**A**) and *IGLL5* **(B)** in normal and KIRC samples. **(C,D)** IHC patterns for *IL2RA* **(C)** and *IGLL5* **(D)** in normal and KIRP samples.

Furthermore, the robustness of *IGLL5* as well as *IL2RA* as biomarkers was verified using primary ccRCC and pRCC samples from Shandong provincial Hospital affiliated to Shandong First University. For ccRCC, IHC images indicated that normal renal tissue had weak staining for *IGLL5* and *IL2RA*, but relatively strong staining patterns for *IGLL5* and *IL2RA* were observed in the cytoplasm of tumor tissues (Figures 6A,B). Moreover, similar staining patterns for *IL2RA* were observed in pRCC tissues and adjacent normal tissues (Figure 6C). However, *IGLL5* staining was weakly positive by IHC, and no significant differences was detected between pRCC tissues and adjacent normal tissues (Figure 6D). These unique IHC staining patterns further confirmed the above results and illustrate that the two genes could be used to predict clinical outcome and distinguish cancerous tissue from normal tissue.

### Pharmacological Analysis

**FIGURE 7:**
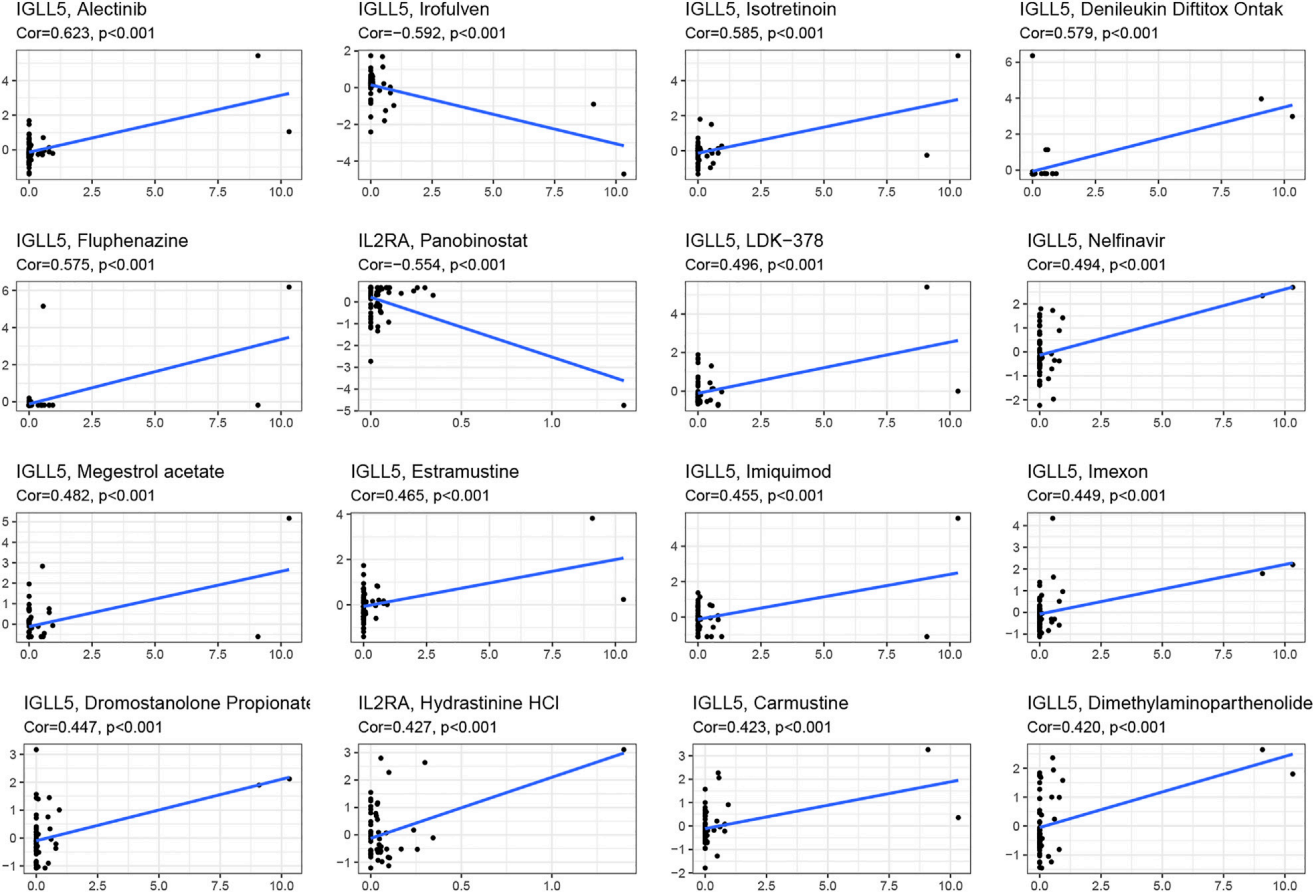
The Prediction analysis between *IGLL5* and *IL2RA* and drug response.

We asked whether selected regulators have ability to predict drug response and potential treatment options for ccRCC and pRCC. Through CellMiner database and Connectivity Map (Cmap), the top 16 relevant correlations between *IGLL5* and *IL2RA* and drug activity were shown in Figure 7. The response to all anticancer drugs was negatively correlated to the expression level of *IL2RA*, including panobinostat, pralatrexate, AT-13387, belinostat, and ponatinib. However, except for irofulven, other anticancer drugs were positively associated with the expression of *IGLL5* (*p* < 0.05, Supplementary Table S1). Moreover, potential drugs for ccRCC and pRCC were predicted based on the top 15 prognosis-related hub genes (Figures 3E,F). The prediction results revealed that 325 and 47 candidate drugs exhibited promising clinical applications for ccRCC and pRCC, respectively (Score ≤ −0.75, Supplementary Table S2, S3). The molecular structure of top 10 potentially active drugs for the treatment of two types of RCC was shown in Supplementary Figure S6.

## Discussion

RCC accounts for more than 90% of kidney cancers, which consists of over 10 histological subtypes. Among these, ccRCC and pRCC are the main subtypes with high incidence and high mortality (Comprehensive molecular c, 2013; Chen et al., 2016; Linehan et al., 2016; Hsieh et al., 2017). Given limited options of effective therapy and modest effects of immunotherapy, there is an urgent need for novel biomarkers for future clinical application and evaluation (Barata and Rini, 2017).

Several studies have established that TME is a potential regulator of cancer progression and a source of therapeutic targets (Quail and Joyce, 2013; Vitale et al., 2019; Ho et al., 2020). In complex TME, immune cells and stromal cells play a significant role in cancer development. Stromal cells are genetically stable, and are considered as promising therapeutic targets for reducing tumor recurrence and drug resistance (Quail and Joyce, 2013). In TME, immune cells are divided into tumor antagonizing and promoting-immune cells. With the ability to regulate tumor progression in various stages (Lei et al., 2020). For example, inflammation could lead to CD8^+^ T cell dysfunction and consequently promote cancer progression (Wherry, 2011). Furthermore, in breast TME, NK cells are immature and dysfunctional NK in hepatocellular TME is related with cancer development (Krneta et al., 2016; Zhang et al., 2017). Garaud et al. found that infiltration of B cells in breast TME was correlated with a favorable prognosis (Garaud et al., 2019). However, several investigations have revealed that B cells may facilitate tumorigenesis by suppressing CD8^+^ T cells and producing cytokines, which contribute to angiogenesis (de Visser et al., 2005; Inoue et al., 2006; Tsou et al., 2016). Currently, the understanding of knowledge of TME of kidney cancers is restricted to specific tumor types and there is a lack of comprehensive analysis.

Here, we used several analyses to explore the TME landscape of ccRCC and pRCC. Firstly, we calculated the immune scores as well as stromal scores and assessed the clinical relevance by integrating of clinical information, which revealed that the survival of ccRCC and pRCC patients was significantly correlated with high immune scores and/or stromal scores. Moreover, various clinical characteristics, such as tumor grade, tumor stage, T stage, M stage were associated with immune scores and stromal scores. Then, by investigating the associated biological functions and pathways *via* GO enrichment and KEGG pathway analyses, we uncovered diverse immune-related functions and pathways, such as B cell mediated immunity, lymphocyte mediated immunity; cytokine-cytokine receptor interaction, chemokine signaling pathways, T cell receptor and B cell receptor signaling pathways. These results are consistent with previous studies and confirmed the essential role of immune cells in ccRCC and pRCC (Xu et al., 2019; Díaz-Montero et al., 2020; Lei et al., 2020; Wan et al., 2020; Zeng et al., 2020). Subsequently, according to STRING database, univariate Cox analysis, and Venn algorithm, we identified the two prognostic hub genes, *IL2RA* and *IGLL5* in ccRCC and pRCC. To further exploit the potential role of these genes, we validated each individual gene in TCGA ccRCC and pRCC cohorts. The results revealed that *IL2RA* is a DEG and is correlated with prognosis of ccRCC and pRCC. However, *IGLL5* was significantly expressed in ccRCC, but not in pRCC. In the ICGC, GEO40912, and GSE2748 validation cohort, we also observed a significantly differential survival trend. However, K-M analysis of OS indicated that high expression of *IL2RA* had a negative impact in GSE2748 dataset, but the difference was not significant. Moreover, the clinical application of the two genes was also validated using primary KIRC and KIRP samples from our hospital, and the results further confirmed the prognostic value of the two genes in clinical application. Previous research on *IGLL5* and *IL2RA* focused predominantly on hematological malignancies. Fusion or mutation of *IGLL5* was identified in Burkitt lymphoma, multiple lymphadenopathy, diffuse large B cell lymphoma, multiple myeloma, and chronic lymphocytic leukemia (Kasar et al., 2015; White et al., 2018; Panea et al., 2019; Li et al., 2020; Mosquera Orgueira et al., 2021). It has been reported that *IGLL5* may act as a risk factor and was related with metastasis and poor prognosis of multiple myeloma (White et al., 2018; Barwick et al., 2019; D'Agostino et al., 2020). *IGLL5* is also correlated with glioblastoma survival and breast cancer, which could serve as a biomarker for relapse-free survival in breast cancer with more than 85% accuracy (Ascierto et al., 2012; Qin et al., 2020). *IL2RA* also exhibits prognostic and diagnostic value in hematopoietic cancers and solid cancers. Elevated expression of *IL2RA* is associated with poor prognosis and may act as a biomarker in acute myeloid leukemia (Du et al., 2019; Nguyen et al., 2019). Up-regulated *IL2RA* was also found in tumor of lung, prostate, breast and melanoma, and thus, could serve as a marker of prognosis and immunoreaction (Kuhn and Dou, 2005).

Based on these results, we explored underlying mechanisms by GSEA analysis and also uncovered various immune-related pathways, further confirming the role of *IGLL5* and *IL2RA* in immune regulation. Afterwards, CIBERSORT indicated that adaptive immune cells account for most of the immune cell infiltrations in ccRCC as well as pRCC. According to difference analysis and correlation analysis, we finally identified the immune cells commonly associated with *IGLL5* and *IL2RA* in ccRCC and pRCC, such as CD8^+^ T cells, Tregs cells and T follicular helper cells, which was consistent with prior studies (Roth et al., 2018; Cañete et al., 2019; Ohue et al., 2019). Considering the unsatisfactory outcomes of therapy, we predicted small molecular compounds and individual response to drugs. Some commonly used drugs (such as oxaliplatin, ponatinib) have been shown to be influenced by *IGLL5* and *IL2RA,* and numerous small molecular compounds have promising potential in future treatments. Currently, drugs targeting *IL2RA* consist of recombinant *IL2*, anti-*IL2RA* antibodies (such as basiliximab, daclizumab), anti-*IL2RA* radioimmunoconjuates (such as CHT-25, ^90^Y-daclizumab), anti-*IL2RA* immunotoxins (such as LMB-2, RFT5-SMPT-dgA) and anti-*IL2RA* antibody drug conjugates (Flynn and Hartley, 2017). However, the efficacy and safety of some drugs is still unclear. Furthermore, *IGLL5*-targeted drugs have not been developed. Thus, further investigation of drugs targeting *IGLL5* and *IL2RA* is of the essence.

In addition, there are other potential factors which also affect clinical outcomes in kidney cancers. Firstly, the spatial distribution of TME-regulated T cells may physically exclude these immune cells from the vicinity of cancer cells. Other essential considerations are that the stages of primary tumor and potentially different constitution of TME at metastatic sites (Fridman et al., 2012). To improve the outcome of ccRCC and pRCC, accurate and efficient biomarkers are indispensable. We investigated the TME and immune-related genes to identify associated immune cells and molecular mechanism as well as clinical value. Our findings suggested that *IGLL5* and *IL2RA* could be used to estimate prognosis and may serve as potential therapeutic targets. The limited number of patients in GEO validation cohort may partially lead to bias. Therefore, in future investigations, expression data and clinical information from our medical center will be collected and further experiments using *in vivo* and *in vitro* models will be implemented to confirm the significant role of the two genes in the TME of kidney cancer.

## Data Availability

The original contributions presented in the study are included in the article/Supplementary Material, further inquiries can be directed to the corresponding authors.

## Nomenclature

Cmap: Connectivity map
ccRCC: Clear cell renal clear cell carcinoma,
DEGs: Differentially expressed genes
ESTIMATE: Estimation of STromal and Immune cells in Malignant Tumours using Expression data
GEO: Gene Expression Omnibus
GO: Gene Ontology
GSEA: Gene set enrichment analysis
ICGC: International Cancer Genome Consortium
KEGG: Kyoto Encyclopedia of Genes and Genomes
pRCC: Papillary renal cell carcinoma
PPI: Protein–protein interaction
RCC: Renal clear cell carcinoma
TCGA: The Cancer Genome Atlas
TME: Tumor microenvironment
IHC: Immunohistochemistry.

## References

[B1] Ascierto M. L. Kmieciak M. Idowu M. O. Manjili R. Zhao Y. Grimes M. (2012). A Signature of Immune Function Genes Associated with Recurrence-free Survival in Breast Cancer Patients. Breast Cancer Res. Treat. 131 (3), 871–880. 10.1007/s10549-011-1470-x 21479927 PMC3431022

[B2] Barata P. C. Rini B. I. (2017). Treatment of Renal Cell Carcinoma: Current Status and Future Directions. CA: a Cancer J. clinicians 67 (6), 507–524. 10.3322/caac.21411 28961310

[B3] Barwick B. G. Neri P. Bahlis N. J. Nooka A. K. Dhodapkar M. V. Jaye D. L. (2019). Multiple Myeloma Immunoglobulin Lambda Translocations Portend Poor Prognosis. Nat. Commun. 10 (1), 1911. 10.1038/s41467-019-09555-6 31015454 PMC6478743

[B4] Cañete P. F. Sweet R. A. Gonzalez-Figueroa P. Papa I. Ohkura N. Bolton H. (2019). Regulatory Roles of IL-10-producing Human Follicular T Cells. J. Exp. Med. 216 (8), 1843–1856. 10.1084/jem.20190493 31209070 PMC6683995

[B5] Chen F. Zhang Y. Şenbabaoğlu Y. Ciriello G. Yang L. Reznik E. (2016). Multilevel Genomics-Based Taxonomy of Renal Cell Carcinoma. Cel Rep. 14 (10), 2476–2489. 10.1016/j.celrep.2016.02.024 PMC4794376 26947078

[B6] Comprehensive Molecular Characterization of clear Cell Renal Cell Carcinoma. Nature. 2013; 499 (7456): 43–49. 10.1038/nature12222 23792563 PMC3771322

[B7] D'Agostino M. Zaccaria G. M. Ziccheddu B. Rustad E. H. Genuardi E. Capra A. (2020). Early Relapse Risk in Patients with Newly Diagnosed Multiple Myeloma Characterized by Next-Generation Sequencing. Clin. Cancer Res. 26 (18), 4832–4841. 10.1158/1078-0432.ccr-20-0951 32616499

[B8] de Visser K. E. Korets L. V. Coussens L. M. (2005). De Novo carcinogenesis Promoted by Chronic Inflammation Is B Lymphocyte Dependent. Cancer cell 7 (5), 411–423. 10.1016/j.ccr.2005.04.014 15894262

[B9] Díaz-Montero C. M. Rini B. I. Finke J. H. (2020). The Immunology of Renal Cell Carcinoma. Nat. Rev. Nephrol. 16 (12), 721–735. 10.1038/s41581-020-0316-3 32733094

[B10] Du W. He J. Zhou W. Shu S. Li J. Liu W. (2019). High IL2RA mRNA Expression Is an Independent Adverse Prognostic Biomarker in Core Binding Factor and Intermediate-Risk Acute Myeloid Leukemia. J. Transl Med. 17 (1), 191. 10.1186/s12967-019-1926-z 31171000 PMC6551869

[B11] Flynn M. J. Hartley J. A. (2017). The Emerging Role of Anti-CD25 Directed Therapies as Both Immune Modulators and Targeted Agents in Cancer. Br. J. Haematol. 179 (1), 20–35. 10.1111/bjh.14770 28556984

[B12] Fridman W. H. Pagès F. Sautès-Fridman C. Galon J. (2012). The Immune Contexture in Human Tumours: Impact on Clinical Outcome. Nat. Rev. Cancer 12 (4), 298–306. 10.1038/nrc3245 22419253

[B13] Garaud S. Buisseret L. Solinas C. Gu-Trantien C. de Wind A. Van den Eynden G. (2019). Tumor Infiltrating B-Cells Signal Functional Humoral Immune Responses in Breast Cancer. JCI insight 5 (18), e129641. 10.1172/jci.insight.129641 PMC6795287 31408436

[B14] Grivennikov S. I. Greten F. R. Karin M. (2010). Immunity, Inflammation, and Cancer. Cell. 140 (6), 883–899. 10.1016/j.cell.2010.01.025 20303878 PMC2866629

[B15] Hanahan D. Coussens L. M. (2012). Accessories to the Crime: Functions of Cells Recruited to the Tumor Microenvironment. Cancer cell 21 (3), 309–322. 10.1016/j.ccr.2012.02.022 22439926

[B16] Ho W. J. Jaffee E. M. Zheng L. (2020). The Tumour Microenvironment in Pancreatic Cancer - Clinical Challenges and Opportunities. Nat. Rev. Clin. Oncol. 17 (9), 527–540. 10.1038/s41571-020-0363-5 32398706 PMC7442729

[B17] Hsieh J. J. Purdue M. P. Signoretti S. Swanton C. Albiges L. Schmidinger M. (2017). Renal Cell Carcinoma. Nat. Rev. Dis. Primers 3, 17009. 10.1038/nrdp.2017.9 28276433 PMC5936048

[B18] Inoue S. Leitner W. W. Golding B. Scott D. (2006). Inhibitory Effects of B Cells on Antitumor Immunity. Cancer Res. 66 (15), 7741–7747. 10.1158/0008-5472.can-05-3766 16885377

[B19] Joyce J. A. Pollard J. W. (2009). Microenvironmental Regulation of Metastasis. Nat. Rev. Cancer 9 (4), 239–252. 10.1038/nrc2618 19279573 PMC3251309

[B20] Kasar S. Kim J. Improgo R. Tiao G. Polak P. Haradhvala N. (2015). Whole-genome Sequencing Reveals Activation-Induced Cytidine Deaminase Signatures during Indolent Chronic Lymphocytic Leukaemia Evolution. Nat. Commun. 6, 8866. 10.1038/ncomms9866 26638776 PMC4686820

[B21] Krneta T. Gillgrass A. Chew M. Ashkar A. A. (2016). The Breast Tumor Microenvironment Alters the Phenotype and Function of Natural Killer Cells. Cell Mol Immunol 13 (5), 628–639. 10.1038/cmi.2015.42 26277898 PMC5037278

[B22] Kuhn D. J. Dou Q. P. (2005). The Role of Interleukin-2 Receptor Alpha in Cancer. Front. Biosci. 10, 1462–1474. 10.2741/1631 15769637

[B23] Lei X. Lei Y. Li J.-K. Du W.-X. Li R.-G. Yang J. (2020). Immune Cells within the Tumor Microenvironment: Biological Functions and Roles in Cancer Immunotherapy. Cancer Lett. 470, 126–133. 10.1016/j.canlet.2019.11.009 31730903

[B24] Li M. Su X. Wang Y. Fan L. Chai J. Li P. (2020). Lineage-negative Lymphoma with a Helper Innate Lymphoid Cell Phenotype. Virchows Arch. 476 (2), 285–293. 10.1007/s00428-019-02658-x 31522287

[B25] Linehan W. M. Linehan W. M. Spellman P. T. Ricketts C. J. Creighton C. J. Fei S. S. (2016). Comprehensive Molecular Characterization of Papillary Renal-Cell Carcinoma. N. Engl. J. Med. 374 (2), 135–145. 10.1056/NEJMoa1505917 26536169 PMC4775252

[B26] Mosquera Orgueira A. Ferreiro Ferro R. Díaz Arias J. Á. Aliste Santos C. Antelo Rodríguez B. Bao Pérez L. (2021). Detection of New Drivers of Frequent B-Cell Lymphoid Neoplasms Using an Integrated Analysis of Whole Genomes. PloS one 16 (5), e0248886. 10.1371/journal.pone.0248886 33945543 PMC8096002

[B27] Nguyen C. H. Glüxam T. Schlerka A. Bauer K. Grandits A. M. Hackl H. (2019). SOCS2 Is Part of a Highly Prognostic 4-gene Signature in AML and Promotes Disease Aggressiveness. Sci. Rep. 9 (1), 9139. 10.1038/s41598-019-45579-0 31235852 PMC6591510

[B28] Ohue Y. Nishikawa H. Regulatory T. (2019). Regulatory T (Treg) Cells in Cancer: Can Treg Cells Be a New Therapeutic Target? Cancer Sci. 110 (7), 2080–2089. 10.1111/cas.14069 31102428 PMC6609813

[B29] Osipov A. Saung M. T. Zheng L. Murphy A. G. (2019). Small Molecule Immunomodulation: the Tumor Microenvironment and Overcoming Immune Escape. J. Immunotherapy Cancer 7 (1), 224. 10.1186/s40425-019-0667-0 PMC6704558 31439034

[B30] Panea R. I. Love C. L. Shingleton J. R. Reddy A. Bailey J. A. Moormann A. M. (2019). The Whole-Genome Landscape of Burkitt Lymphoma Subtypes. Blood 134 (19), 1598–1607. 10.1182/blood.2019001880 31558468 PMC6871305

[B31] Pitt J. M. Marabelle A. Eggermont A. Soria J.-C. Kroemer G. Zitvogel L. (2016). Targeting the Tumor Microenvironment: Removing Obstruction to Anticancer Immune Responses and Immunotherapy. Ann. Oncol. 27 (8), 1482–1492. 10.1093/annonc/mdw168 27069014

[B32] Qin Z. Zhang X. Chen Z. Liu N. (2020). Establishment and Validation of an Immune-Based Prognostic Score Model in Glioblastoma. Int. immunopharmacology 85, 106636. 10.1016/j.intimp.2020.106636 32534425

[B33] Quail D. F. Joyce J. A. (2013). Microenvironmental Regulation of Tumor Progression and Metastasis. Nat. Med. 19 (11), 1423–1437. 10.1038/nm.3394 24202395 PMC3954707

[B34] Ravaud A. Motzer R. J. Pandha H. S. George D. J. Pantuck A. J. Patel A. (2016). Adjuvant Sunitinib in High-Risk Renal-Cell Carcinoma after Nephrectomy. N. Engl. J. Med. 375 (23), 2246–2254. 10.1056/nejmoa1611406 27718781

[B35] Roth T. L. Puig-Saus C. Yu R. Shifrut E. Carnevale J. Li P. J. (2018). Reprogramming Human T Cell Function and Specificity with Non-viral Genome Targeting. Nature 559 (7714), 405–409. 10.1038/s41586-018-0326-5 29995861 PMC6239417

[B36] Siegel R. L. Miller K. D. Fuchs H. E. JemalCancer Statistics A. (2021). Cancer Statistics, 2021. CA A. Cancer J. Clin. 71 (1), 7–33. 10.3322/caac.21654 33433946

[B37] Steffens S. Janssen M. Roos F. C. Becker F. Schumacher S. Seidel C. (2012). Incidence and Long-Term Prognosis of Papillary Compared to clear Cell Renal Cell Carcinoma - A Multicentre Study. Eur. J. Cancer 48 (15), 2347–2352. 10.1016/j.ejca.2012.05.002 22698386

[B38] Szklarczyk D. Gable A. L. Lyon D. Junge A. Wyder S. Huerta-Cepas J. (2019). STRING V11: Protein-Protein Association Networks with Increased Coverage, Supporting Functional Discovery in Genome-wide Experimental Datasets. Nucleic Acids Res. 47 (D1), D607–D613. 10.1093/nar/gky1131 30476243 PMC6323986

[B39] Tsou P. Katayama H. Ostrin E. J. Hanash S. M. (2016). The Emerging Role of B Cells in Tumor Immunity. Cancer Res. 76 (19), 5597–5601. 10.1158/0008-5472.can-16-0431 27634765

[B40] Vitale I. Manic G. Coussens L. M. Kroemer G. Galluzzi L. (2019). Macrophages and Metabolism in the Tumor Microenvironment. Cel Metab. 30 (1), 36–50. 10.1016/j.cmet.2019.06.001 31269428

[B41] Vuong L. Kotecha R. R. Voss M. H. Hakimi A. A. (2019). Tumor Microenvironment Dynamics in Clear-Cell Renal Cell Carcinoma. Cancer Discov. 9 (10), 1349–1357. 10.1158/2159-8290.cd-19-0499 31527133 PMC6774890

[B42] Wan B. Liu B. Huang Y. Lv C. (2020). Identification of Genes of Prognostic Value in the ccRCC Microenvironment from TCGA Database. Mol. Genet. Genomic Med. 8 (4), e1159. 10.1002/mgg3.1159 32012488 PMC7196483

[B43] Wang Q. Chen C. Ding Q. Zhao Y. Wang Z. Chen J. (2020). METTL3-mediated m6A Modification of HDGF mRNA Promotes Gastric Cancer Progression and Has Prognostic Significance. Gut 69 (7), 1193–1205. 10.1136/gutjnl-2019-319639 31582403

[B44] Wherry E. J. (2011). T Cell Exhaustion. Nat. Immunol. 12 (6), 492–499. 10.1038/ni.2035 21739672

[B45] White B. S. Lanc I. O’Neal J. Gupta H. Fulton R. S. Schmidt H. (2018). A Multiple Myeloma-specific Capture Sequencing Platform Discovers Novel Translocations and Frequent, Risk-Associated point Mutations in IGLL5. Blood Cancer J. 8 (3), 35. 10.1038/s41408-018-0062-y 29563506 PMC5862875

[B46] Wu T. Dai Y. (2017). Tumor Microenvironment and Therapeutic Response. Cancer Lett. 387, 61–68. 10.1016/j.canlet.2016.01.043 26845449

[B47] Xu W.-H. Xu Y. Wang J. Wan F.-N. Wang H.-K. Cao D.-L. (2019). Prognostic Value and Immune Infiltration of Novel Signatures in clear Cell Renal Cell Carcinoma Microenvironment. Aging 11 (17), 6999–7020. 10.18632/aging.102233 31493764 PMC6756904

[B48] Yoshihara K. Shahmoradgoli M. Martínez E. Vegesna R. Kim H. Torres-Garcia W. (2013). Inferring Tumour Purity and Stromal and Immune Cell Admixture from Expression Data. Nat. Commun. 4, 2612. 10.1038/ncomms3612 24113773 PMC3826632

[B49] Zeng Q. Zhang W. Li X. Lai J. Li Z. (2020). Bioinformatic Identification of Renal Cell Carcinoma Microenvironment-Associated Biomarkers with Therapeutic and Prognostic Value. Life Sci. 243, 117273. 10.1016/j.lfs.2020.117273 31926244

[B50] Zhang Q.-F. Yin W.-W. Xia Y. Yi Y.-Y. He Q.-F. Wang X. (2017). Liver-infiltrating CD11b−CD27− NK Subsets Account for NK-Cell Dysfunction in Patients with Hepatocellular Carcinoma and Are Associated with Tumor Progression. Cel Mol Immunol 14 (10), 819–829. 10.1038/cmi.2016.28 PMC5649104 27321064

