# Supplementary figures and images for: Integrative Analysis of Immune-Related Genes in the Tumor Microenvironment of Renal Clear Cell Carcinoma and Renal Papillary Cell Carcinoma
Source: Front Mol Biosci. 2021 Nov 23;8:760031. doi: 10.3389/fmolb.2021.760031 (PMC8650138; doi:10.3389/fmolb.2021.760031)

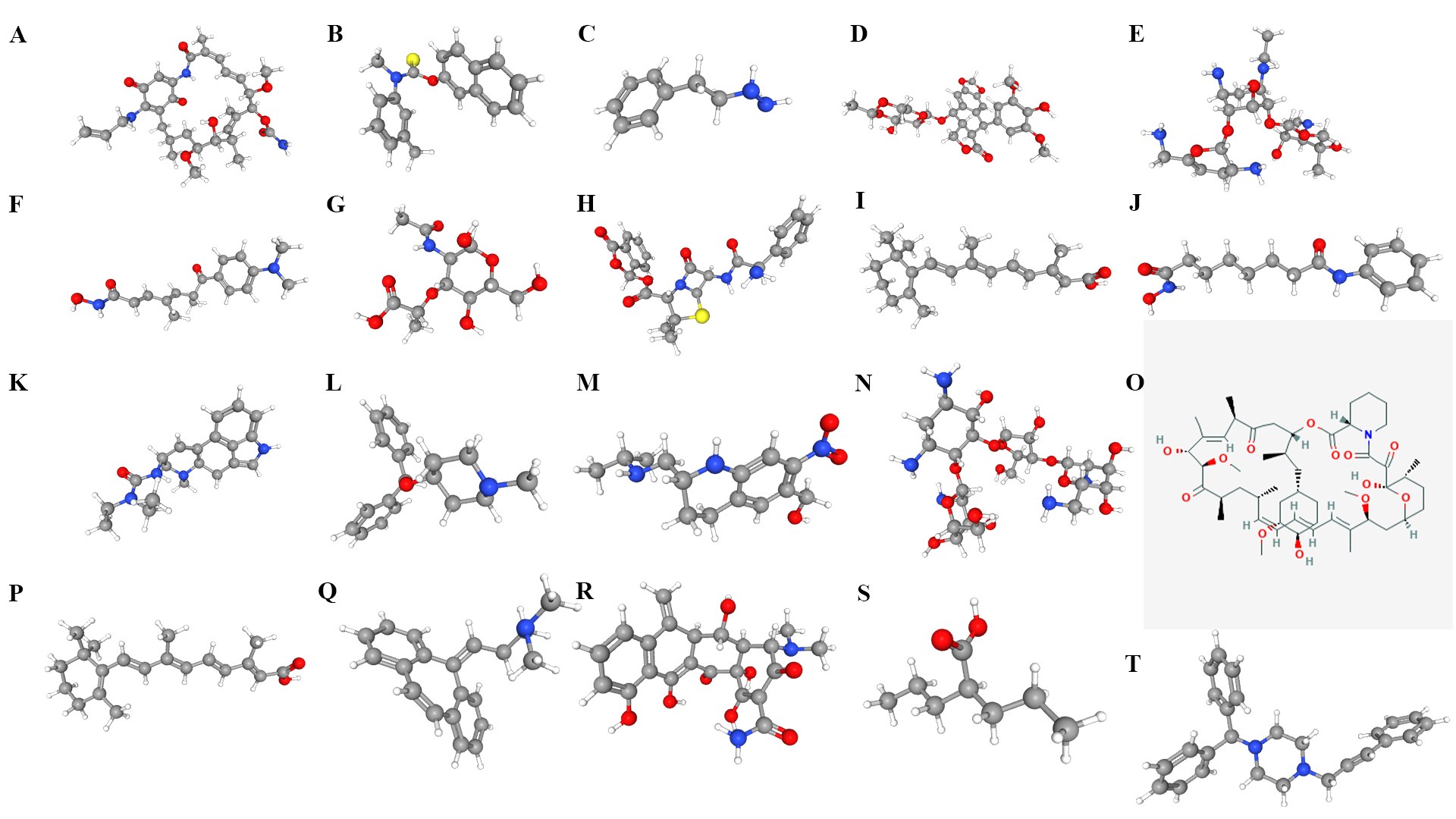

Supplement: Supplementary file 1 [file Image6.TIF]

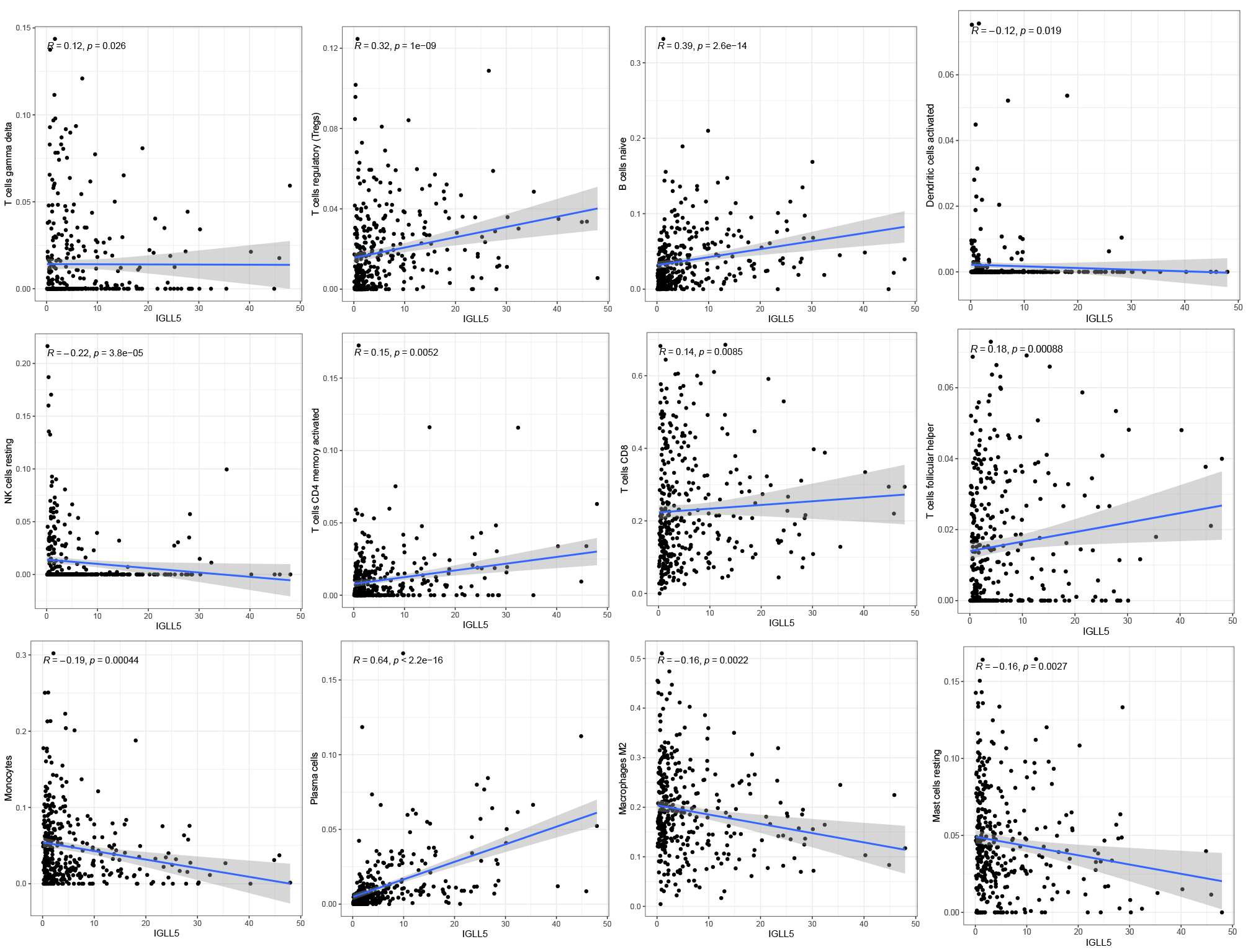

Supplement: Supplementary file 2 [file Image3.TIF]

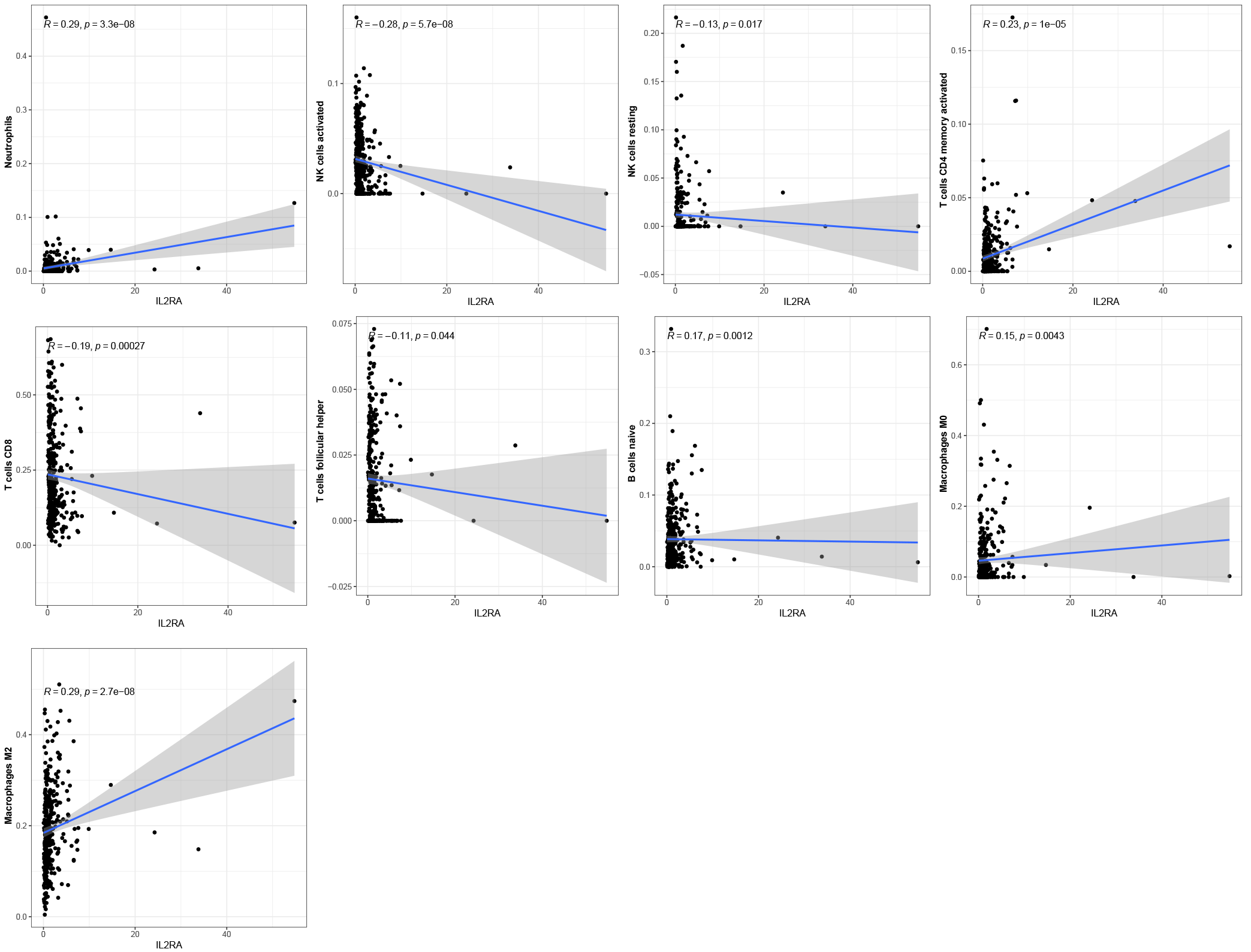

Supplement: Supplementary file 3 [file Image4.TIF]

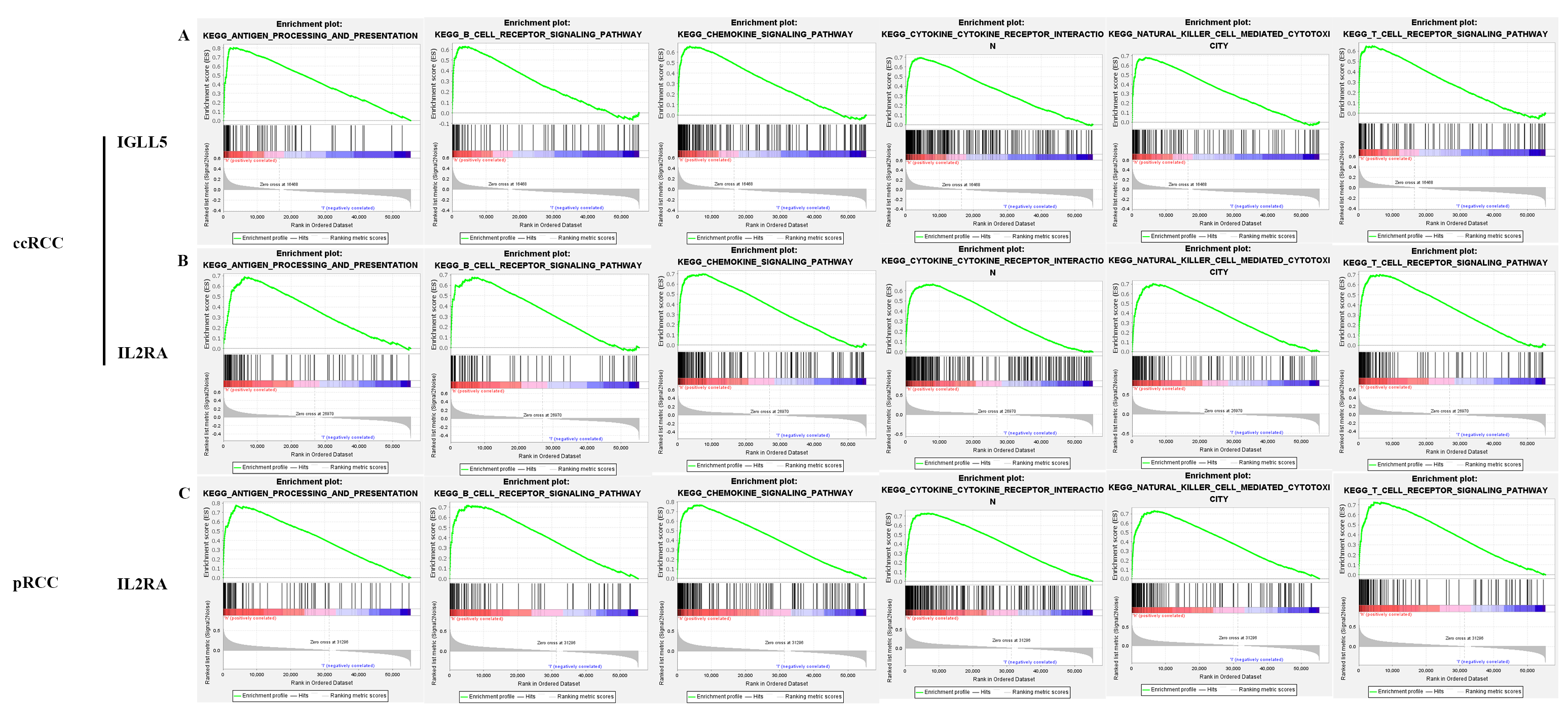

Supplement: Supplementary file 4 [file Image2.TIF]

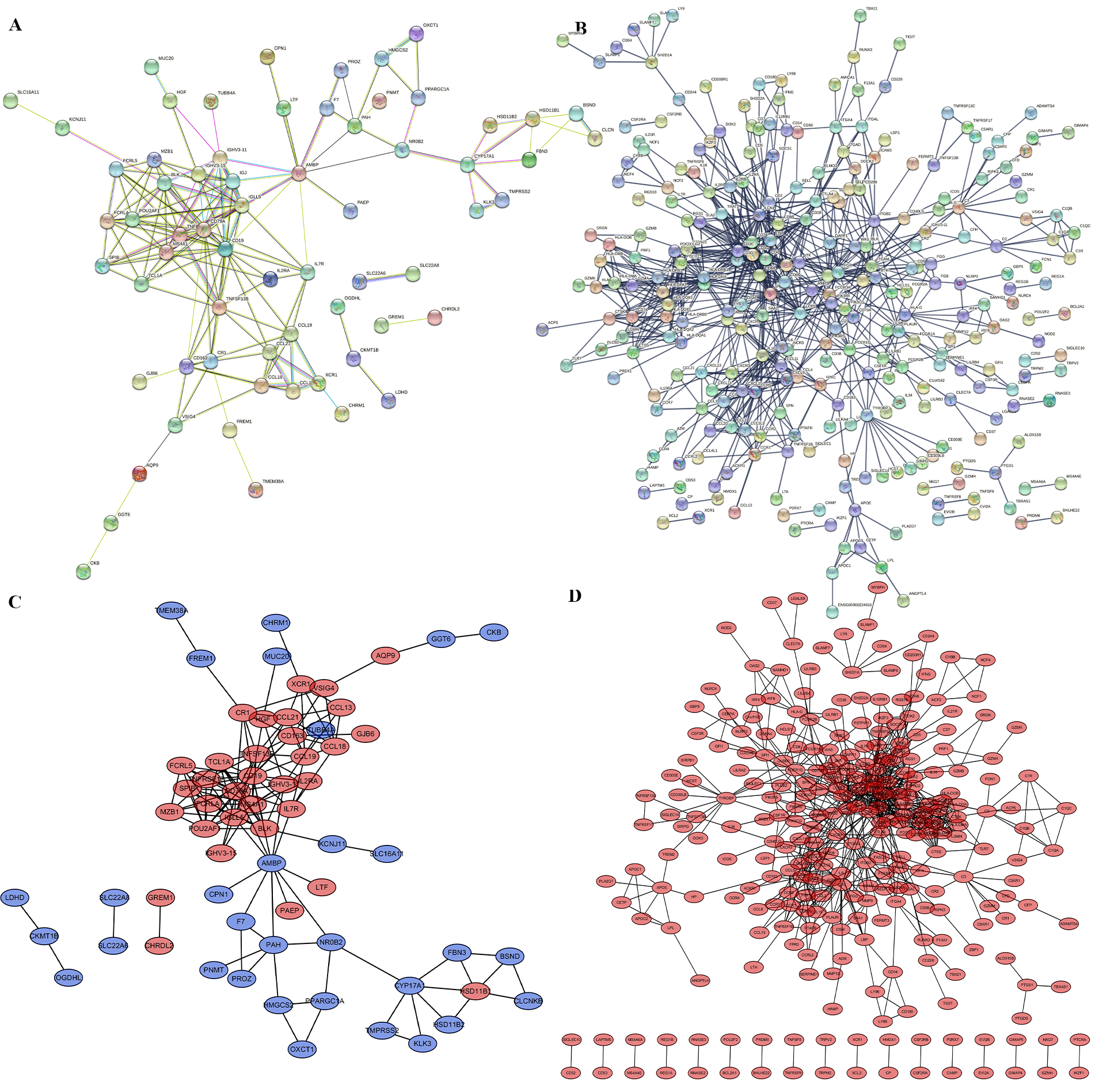

Supplement: Supplementary file 5 [file Image1.TIF]

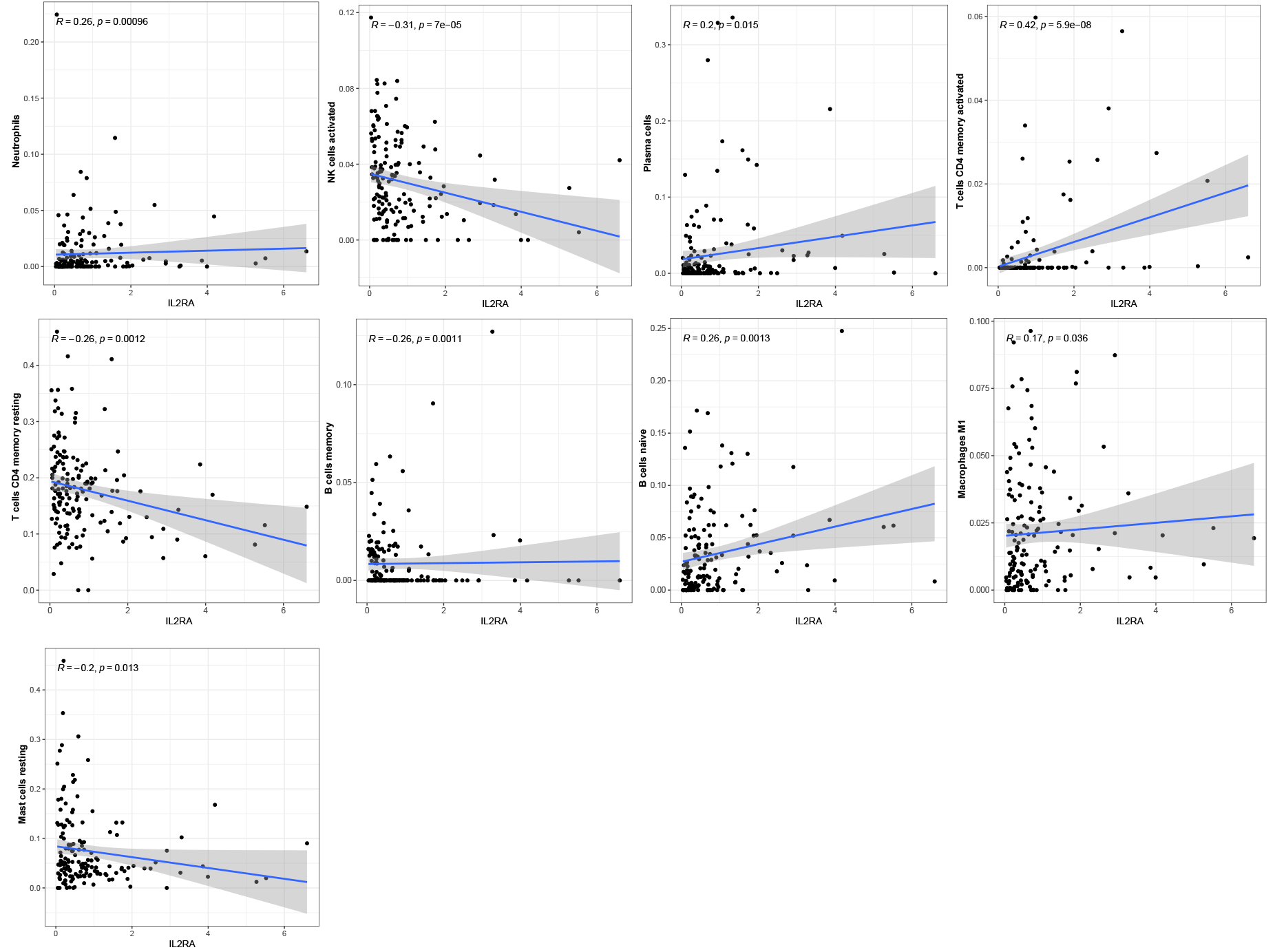

Supplement: Supplementary file 7 [file Image5.TIF]
