# Supplementary material for: Integrative Analysis of Immune-Related Genes in the Tumor Microenvironment of Renal Clear Cell Carcinoma and Renal Papillary Cell Carcinoma
Source: Front Mol Biosci. 2021 Nov 23;8:760031. doi: 10.3389/fmolb.2021.760031 (PMC8650138; doi:10.3389/fmolb.2021.760031)
Supplement: Supplementary file 6 [file DataSheet1.PDF]

Supplementary Material

1 Supplementary Figures and Tables

Supplementary Table 1. The correlation between drug response and *IGLL2* and *IL2RA* expression level.

Gene	Drug	Cor	*p*-value
IGLL5	Irofulven	-0.59156	6.45E-07
IL2RA	Panobinostat	-0.55435	4.32E-06
IL2RA	Pralatrexate	-0.378	0.002904
IL2RA	AT-13387	-0.33934	0.007991
IL2RA	Belinostat	-0.2736	0.034405
IL2RA	Ponatinib	-0.2692	0.037535
IGLL5	Chlorambucil	0.254281	0.049929
IGLL5	6-Mercaptopurine	0.25706	0.047395
IGLL5	Pipamperone	0.258706	0.045945
IGLL5	Fulvestrant	0.262306	0.0429
IGLL5	Crizotinib	0.266595	0.039492
IGLL5	XK-469	0.266676	0.03943
IGLL5	SR16157	0.26822	0.038261
IGLL5	Salinomycin	0.27325	0.034649
IGLL5	Itraconazole	0.277254	0.03198
IGLL5	Palbociclib	0.286011	0.026737
IGLL5	Nitrogen mustard	0.286715	0.02635
IGLL5	Arsenic trioxide	0.290954	0.024111
IGLL5	Raltitrexed	0.297075	0.021165
IGLL5	Hydroxyurea	0.297107	0.02115
IGLL5	Calusterone	0.300762	0.019542
IGLL5	LMP-400	0.301068	0.019412
IGLL5	Parthenolide	0.304343	0.018068
IGLL5	Belinostat	0.308867	0.016342
IGLL5	Valrubicin	0.308994	0.016296
IGLL5	Epirubicin	0.311699	0.015336
IGLL5	Teniposide	0.318281	0.013199
IGLL5	Raloxifene	0.321968	0.012118
IGLL5	Ifosfamide	0.322488	0.011972
IGLL5	Tegafur	0.323452	0.011705
IGLL5	Lomustine	0.350659	0.006017
IGLL5	Oxaliplatin	0.354526	0.005448
IGLL5	AP-26113	0.359572	0.004777
IGLL5	Elesclomol	0.378145	0.002892
IGLL5	Celecoxib	0.380064	0.002742
IGLL5	Ethinyl estradiol	0.381266	0.002651
IGLL5	Etoposide	0.386212	0.002305

IGLL5	Entinostat	0.416429	0.000935
IGLL5	7-Hydroxystaurosporine	0.41796	0.000891
IGLL5	Dimethylaminoparthenolide	0.420349	0.000826
IGLL5	Carmustine	0.42255	0.00077
IL2RA	Hydrastinine HCl	0.426993	0.000668
IGLL5	Dromostanolone Propionate	0.446543	0.000348
IGLL5	Imexon	0.449271	0.000317
IGLL5	Imiquimod	0.455174	0.000258
IGLL5	Estramustine	0.464787	0.000183
IGLL5	Megestrol acetate	0.481501	9.83E-05
IGLL5	Nelfinavir	0.494169	6.00E-05
IGLL5	LDK-378	0.496025	5.57E-05
IGLL5	Fluphenazine	0.57519	1.53E-06
IGLL5	Denileukin Diftitox Ontak	0.579013	1.26E-06
IGLL5	Isotretinoin	0.584937	9.20E-07
IGLL5	Alectinib	0.622786	1.08E-07

Supplementary Table 2. The 325 potential drugs for ccRCC treatment.

Drug name	Dose	Cell	Score	Instance ID
tanespimycin	100 nM	MCF7	-1	221
tolnaftate	13 µM	HL60	-0.986	2001
phenelzine	17 µM	PC3	-0.956	3802
etoposide	7 µM	PC3	-0.928	6681
netilmicin	3 µM	MCF7	-0.925	3524
trichostatin A	100 nM	PC3	-0.925	1891
N-acetylmuramic acid	14 µM	HL60	-0.921	1326
talampicillin	8 µM	MCF7	-0.919	3515
tretinoin	1 µM	MCF7	-0.913	224
vorinostat	10 µM	HL60	-0.913	6179
asiaticoside	4 µM	MCF7	-0.91	3504
LY-294002	10 µM	MCF7	-0.9	5236
zoxazolamine	24 µM	MCF7	-0.898	2625
LY-294002	10 µM	MCF7	-0.898	1016
solanine	5 µM	PC3	-0.898	4087
racecadotril	10 µM	PC3	-0.897	5755
heliotrine	13 µM	PC3	-0.896	3717
aconitine	6 µM	HL60	-0.886	1784
isoxicam	12 µM	HL60	-0.885	1862
valproic acid	50 µM	MCF7	-0.885	6982
paclitaxel	5 µM	MCF7	-0.881	5320
piroxicam	12 µM	HL60	-0.881	1405
clozapine	10 µM	MCF7	-0.88	6988
flupentixol	8 µM	MCF7	-0.88	5307
PHA-00767505E	10 µM	PC3	-0.879	6545

etiocholanolone	14 µM	PC3	-0.878	4298
alvespimycin	100 nM	HL60	-0.877	1154
hexestrol	15 µM	HL60	-0.877	2515
novobiocin	6 µM	MCF7	-0.876	4392
AG-013608	10 µM	MCF7	-0.876	6395
hydrochlorothiazide	13 µM	PC3	-0.876	1906
phensuximide	21 µM	MCF7	-0.875	3521
sirolimus	100 nM	MCF7	-0.875	5204
pridinol	10 µM	HL60	-0.875	2715
tacrolimus	1 µM	MCF7	-0.872	378
citalopram	1 µM	PC3	-0.872	4555
suxibuzone	9 µM	HL60	-0.872	2503
isoxicam	12 µM	MCF7	-0.87	1698
metolazone	11 µM	MCF7	-0.869	5392
alvespimycin	100 nM	MCF7	-0.867	5210
valproic acid	50 µM	MCF7	-0.867	1002
colecalciferol	10 µM	HL60	-0.866	2436
vorinostat	10 µM	MCF7	-0.866	1000
sulfacetamide	16 µM	HL60	-0.865	1859
trichostatin A	100 nM	MCF7	-0.864	5693
metrifonate	16 µM	HL60	-0.864	1839
lymecycline	7 µM	MCF7	-0.862	3514
metixene	12 µM	PC3	-0.861	6672
suloctidil	12 µM	MCF7	-0.86	5021
verteporfin	3 µM	HL60	-0.859	6133
estradiol	10 nM	PC3	-0.859	665
tanespimycin	1 µM	MCF7	-0.859	998
isocorydine	12 µM	MCF7	-0.855	2780
fusaric acid	22 µM	HL60	-0.855	1308
amiprilose	12 µM	MCF7	-0.855	4119
pentetrazol	29 µM	MCF7	-0.853	5508
monorden	100 nM	MCF7	-0.853	1057
ivermectin	5 µM	PC3	-0.853	5853
sulpiride	12 µM	HL60	-0.853	1967
gibberellic acid	12 µM	MCF7	-0.852	4818
nomifensine	11 µM	PC3	-0.852	5863
aztreonam	9 µM	PC3	-0.851	5110
mimosine	20 µM	PC3	-0.851	6703
berberine	11 µM	MCF7	-0.85	2770
estradiol	100 nM	MCF7	-0.85	6968
iobenguane	11 µM	MCF7	-0.849	2878
pyrantel	11 µM	MCF7	-0.848	5513
rolitetracycline	8 µM	PC3	-0.846	6731
propoxycaine	12 µM	MCF7	-0.845	3583
tiratricol	6 µM	HL60	-0.844	1412
kawain	17 µM	MCF7	-0.843	4693
ornidazole	18 µM	MCF7	-0.843	2272
dyclonine	12 µM	HL60	-0.843	2392

iohexol	5 µM	PC3	-0.843	4643
haloperidol	11 µM	MCF7	-0.842	1539
genistein	10 µM	PC3	-0.841	4459
dexamethasone	9 µM	PC3	-0.84	5797
propofol	22 µM	HL60	-0.84	3048
cytisine	21 µM	HL60	-0.838	1766
salbutamol	17 µM	MCF7	-0.838	4700
isoxicam	12 µM	PC3	-0.837	1820
bupivacaine	12 µM	HL60	-0.837	2404
sulfadiazine	16 µM	MCF7	-0.836	1688
aztreonam	9 µM	HL60	-0.836	1435
nefopam	14 µM	PC3	-0.835	3730
betonicine	25 µM	PC3	-0.835	3745
calcium pantothenate	8 µM	PC3	-0.835	4189
bemegride	26 µM	HL60	-0.834	3051
trichostatin A	100 nM	MCF7	-0.833	1637
estropipate	9 µM	MCF7	-0.832	6068
PNU-0251126	1 µM	PC3	-0.832	3692
aminophylline	10 µM	HL60	-0.832	3036
calcium folinate	8 µM	PC3	-0.831	7401
colchicine	10 µM	HL60	-0.831	1598
clonidine	15 µM	PC3	-0.831	4478
nifenazone	13 µM	PC3	-0.831	2122
Prestwick-860	35 µM	HL60	-0.83	3040
colforsin	50 µM	MCF7	-0.83	913
imipramine	13 µM	PC3	-0.83	1807
tobramycin	9 µM	PC3	-0.83	4081
emetine	7 µM	MCF7	-0.829	2801
0173570-0000	10 µM	MCF7	-0.829	4712
trimethoprim	14 µM	PC3	-0.829	7377
cloxacillin	9 µM	PC3	-0.829	2126
theobromine	22 µM	PC3	-0.828	6613
cefotaxime	8 µM	PC3	-0.828	5830
alverine	8 µM	PC3	-0.828	6345
metformin	24 µM	MCF7	-0.827	1694
3-aminobenzamide	10 mM	MCF7	-0.826	590
L-methionine sulfoximine	22 µM	PC3	-0.826	4070
monorden	100 nM	PC3	-0.826	4443
riluzole	15 µM	PC3	-0.825	7365
atractyloside	5 µM	PC3	-0.825	7393
meprylcaine	15 µM	PC3	-0.824	5723
16-phenyltetranorprostaglandin E2	10 µM	PC3	-0.824	7546
isocarboxazid	17 µM	PC3	-0.824	7383
tomelukast	1 µM	MCF7	-0.824	222
carbamazepine	17 µM	PC3	-0.823	5093
pindolol	16 µM	MCF7	-0.823	6834
Prestwick-689	10 µM	PC3	-0.823	5816

papaverine	11 µM	HL60	-0.823	1755
indapamide	11 µM	HL60	-0.823	2361
mycophenolic acid	12 µM	MCF7	-0.822	4137
IC-86621	1 µM	PC3	-0.819	7518
gemfibrozil	16 µM	MCF7	-0.818	2277
prochlorperazine	10 µM	PC3	-0.818	4439
Prestwick-689	10 µM	MCF7	-0.818	7173
aztreonam	9 µM	MCF7	-0.818	2282
BCB000038	10 µM	PC3	-0.817	7520
tretinoin	13 µM	HL60	-0.817	1548
tamoxifen	1 µM	MCF7	-0.817	380
BCB000038	10 µM	PC3	-0.817	7547
cobalt chloride	100 µM	MCF7	-0.816	379
chlorcyclizine	12 µM	PC3	-0.816	4546
dipyridamole	8 µM	HL60	-0.816	2017
prochlorperazine	10 µM	MCF7	-0.816	5212
Prestwick-984	9 µM	MCF7	-0.816	4948
sulfamerazine	15 µM	PC3	-0.814	3718
LY-294002	10 µM	ssMCF7	-0.814	401
valinomycin	100 nM	PC3	-0.814	5911
terazosin	9 µM	HL60	-0.813	2530
remoxipride	10 µM	HL60	-0.813	3124
mimosine	20 µM	MCF7	-0.812	2638
pentamidine	7 µM	HL60	-0.811	2473
chlorhexidine	8 µM	HL60	-0.81	2025
chlorhexidine	8 µM	PC3	-0.809	6302
docosahexaenoic acid ethyl ester	100 µM	PC3	-0.808	664
ticarcillin	9 µM	MCF7	-0.808	7185
dacarbazine	22 µM	HL60	-0.807	1762
PNU-0230031	10 µM	MCF7	-0.806	4754
sulindac	11 µM	MCF7	-0.805	1693
valproic acid	200 µM	PC3	-0.805	4438
betulin	9 µM	MCF7	-0.804	3513
naftopidil	9 µM	PC3	-0.804	4193
molindone	13 µM	PC3	-0.804	4199
nabumetone	18 µM	HL60	-0.804	3108
ursolic acid	9 µM	PC3	-0.804	2067
NS-398	10 µM	MCF7	-0.803	6897
suramin sodium	10 µM	MCF7	-0.802	7496
prochlorperazine	7 µM	MCF7	-0.802	5010
cefaclor	10 µM	HL60	-0.801	2483
brinzolamide	10 µM	MCF7	-0.801	3230
cycloserine	39 µM	PC3	-0.8	7134
proguanil	14 µM	MCF7	-0.8	3505
CP-944629	10 µM	MCF7	-0.8	7544
troglitazone	10 µM	MCF7	-0.8	5229
fosfosal	18 µM	MCF7	-0.8	4823
harmalol	15 µM	MCF7	-0.799	2892

kinetin	19 µM	MCF7	-0.798	6813
cinchonine	14 µM	HL60	-0.798	2133
AH-23848	1 µM	MCF7	-0.798	6903
cefapirin	9 µM	PC3	-0.798	7142
etynodiol	10 µM	MCF7	-0.798	5024
idoxuridine	11 µM	PC3	-0.797	4200
antazoline	13 µM	PC3	-0.797	7128
ticarcillin	9 µM	HL60	-0.797	6146
azacyclonol	15 µM	MCF7	-0.797	5398
HC toxin	100 nM	MCF7	-0.797	909
trichostatin A	1 µM	MCF7	-0.796	7499
flumetasone	10 µM	MCF7	-0.796	4734
tanespimycin	1 µM	MCF7	-0.795	1631
tridihexethyl	11 µM	MCF7	-0.795	3526
15-delta prostaglandin J2	10 µM	MCF7	-0.795	1069
lymecycline	7 µM	MCF7	-0.795	5994
etodolac	14 µM	HL60	-0.795	1407
demeclocycline	8 µM	PC3	-0.794	3706
tetryzoline	17 µM	HL60	-0.794	2507
Prestwick-981	11 µM	MCF7	-0.794	6504
naphazoline	16 µM	PC3	-0.794	6604
famotidine	12 µM	PC3	-0.794	1946
cefalexin	11 µM	HL60	-0.794	1273
riluzole	15 µM	MCF7	-0.793	4689
fenbendazole	13 µM	MCF7	-0.793	3888
dirithromycin	5 µM	MCF7	-0.793	2863
flunisolide	9 µM	MCF7	-0.793	3828
loperamide	8 µM	MCF7	-0.793	5632
practolol	15 µM	HL60	-0.793	1587
cyclic adenosine monophosphate	12 µM	MCF7	-0.793	3531
pyrimethamine	16 µM	HL60	-0.792	1974
ketoprofen	16 µM	PC3	-0.792	3729
trifluoperazine	10 µM	PC3	-0.792	4448
nifedipine	10 µM	MCF7	-0.792	603
clotrimazole	12 µM	MCF7	-0.792	3166
thioproperazine	6 µM	MCF7	-0.792	2236
clotrimazole	50 µM	MCF7	-0.791	905
thioperamide	10 µM	PC3	-0.791	4675
moracizine	9 µM	MCF7	-0.79	3520
articaine	12 µM	HL60	-0.789	3138
solanine	5 µM	HL60	-0.789	2152
sulfaphenazole	13 µM	MCF7	-0.789	1673
haloperidol	10 µM	PC3	-0.789	4468
acetazolamide	18 µM	HL60	-0.789	1850
isoetarine	12 µM	HL60	-0.788	2711
flumequine	15 µM	HL60	-0.788	1429
SR-95639A	10 µM	MCF7	-0.788	4977

BAS-012416453	38 µM	MCF7	-0.787	6880
thiamphenicol	11 µM	MCF7	-0.786	1704
5213008	18 µM	MCF7	-0.786	898
glibenclamide	8 µM	MCF7	-0.785	3163
dexamethasone	9 µM	PC3	-0.785	2079
valproic acid	500 µM	HL60	-0.785	6199
mebhydrolin	5 µM	MCF7	-0.785	3269
benzydamine	12 µM	MCF7	-0.785	7169
ioxaglic acid	3 µM	MCF7	-0.785	3528
acebutolol	11 µM	PC3	-0.784	6631
trichostatin A	100 nM	HL60	-0.784	2523
tanespimycin	1 µM	MCF7	-0.783	1063
tanespimycin	1 µM	MCF7	-0.783	5223
cinnarizine	11 µM	MCF7	-0.783	7174
yohimbic acid	11 µM	PC3	-0.782	4082
dequalinium chloride	8 µM	MCF7	-0.782	5396
(+)-isoprenaline	11 µM	HL60	-0.782	3046
harmalol	15 µM	PC3	-0.781	5076
lobelanidine	11 µM	HL60	-0.781	1747
pyrimethamine	16 µM	PC3	-0.781	4194
geldanamycin	1 µM	MCF7	-0.78	5225
clotrimazole	12 µM	HL60	-0.78	1549
physostigmine	6 µM	PC3	-0.78	5749
5286656	50 µM	MCF7	-0.78	889
isoxsuprine	12 µM	HL60	-0.779	1985
benzathine benzylpenicillin	4 µM	HL60	-0.779	2939
ioxaglic acid	3 µM	MCF7	-0.778	7470
trichostatin A	100 nM	MCF7	-0.778	1672
suprofen	15 µM	MCF7	-0.777	4123
guanethidine	13 µM	MCF7	-0.777	3171
LY-294002	10 µM	MCF7	-0.777	1065
amitriptyline	13 µM	PC3	-0.777	6353
dicloxacillin	8 µM	PC3	-0.775	6666
5155877	10 µM	PC3	-0.775	6569
colistin	3 µM	MCF7	-0.775	4796
geldanamycin	1 µM	HL60	-0.775	1169
mafenide	18 µM	PC3	-0.774	2124
LY-294002	10 µM	MCF7	-0.774	5233
trichostatin A	1 µM	MCF7	-0.774	5231
mifepristone	9 µM	MCF7	-0.774	7183
clenbuterol	13 µM	MCF7	-0.773	5266
fluvastatin	9 µM	PC3	-0.773	6691
fulvestrant	1 µM	HL60	-0.773	1146
pimethixene	10 µM	MCF7	-0.773	7426
AR-A014418	10 µM	PC3	-0.773	7097
fulvestrant	1 µM	PC3	-0.772	7096
lomustine	100 µM	PC3	-0.772	7094
dyclonine	12 µM	MCF7	-0.771	7022

xylometazoline	14 µM	MCF7	-0.771	7020
alpha-estradiol	10 nM	HL60	-0.771	1151
tanespimycin	1 µM	MCF7	-0.771	947
diethylstilbestrol	15 µM	PC3	-0.77	4547
epiandrosterone	14 µM	HL60	-0.769	2444
dicycloverine	12 µM	PC3	-0.769	4581
alpha-ergocryptine	7 µM	MCF7	-0.769	3434
loxapine	9 µM	MCF7	-0.769	5293
isoetarine	12 µM	MCF7	-0.768	7170
sulpiride	12 µM	MCF7	-0.768	4389
atropine oxide	12 µM	MCF7	-0.768	6812
5224221	12 µM	MCF7	-0.768	839
diprophylline	16 µM	PC3	-0.767	5063
dihydroergotamine	3 µM	PC3	-0.767	2081
aceclofenac	11 µM	PC3	-0.767	2117
cefuroxime	9 µM	HL60	-0.766	2526
gentamicin	3 µM	PC3	-0.766	2082
5140203	15 µM	MCF7	-0.766	908
ondansetron	12 µM	MCF7	-0.766	3575
lidoflazine	8 µM	PC3	-0.766	5804
2,6-dimethylpiperidine	27 µM	MCF7	-0.765	6049
pyrazinamide	32 µM	PC3	-0.765	6617
josamycin	5 µM	PC3	-0.765	4631
amiloride	13 µM	HL60	-0.764	1970
isradipine	11 µM	HL60	-0.764	3129
AG-013608	10 µM	PC3	-0.764	5909
terbutaline	7 µM	PC3	-0.763	5764
triprolidine	13 µM	MCF7	-0.763	7408
dihydroergotamine	3 µM	MCF7	-0.762	6840
lysergol	16 µM	MCF7	-0.761	3261
terbutaline	7 µM	MCF7	-0.761	3202
bucladesine	2 µM	MCF7	-0.761	842
merbromin	5 µM	MCF7	-0.76	4722
dropropizine	17 µM	MCF7	-0.759	5531
nystatin	4 µM	MCF7	-0.759	4807
nifuroxazide	15 µM	MCF7	-0.759	4835
oxymetazoline	13 µM	MCF7	-0.758	2278
5230742	17 µM	MCF7	-0.758	862
sulfaquinoxaline	12 µM	HL60	-0.758	2528
etofenamate	11 µM	MCF7	-0.758	4108
troglitazone	10 µM	PC3	-0.758	462
butacaine	13 µM	PC3	-0.757	5748
triamterene	16 µM	HL60	-0.757	1861
tanespimycin	1 µM	MCF7	-0.757	1650
cloxacillin	9 µM	HL60	-0.757	1443
sirolimus	100 nM	HL60	-0.756	2667
hecogenin	9 µM	MCF7	-0.756	7175

carbamazepine	17 µM	MCF7	-0.756	5518
dextromethorphan	11 µM	HL60	-0.756	1281
acetazolamide	18 µM	PC3	-0.755	1808
ciprofloxacin	11 µM	MCF7	-0.755	5299
oxolinic acid	15 µM	PC3	-0.755	5094
bisacodyl	11 µM	HL60	-0.754	2435
astemizole	9 µM	MCF7	-0.753	6807
dienestrol	15 µM	MCF7	-0.752	6208
meteneprost	10 µM	MCF7	-0.752	7500
flunixin	8 µM	PC3	-0.752	3713
4-hydroxyphenazone	20 µM	HL60	-0.752	1997
5151277	14 µM	MCF7	-0.752	903
cinchonine	14 µM	MCF7	-0.751	4107
Prestwick-972	23 µM	PC3	-0.751	7266
metrifonate	16 µM	MCF7	-0.751	1675

Supplementary Table 3. The 47 potential drugs for pRCC treatment.

Drug name	Dose	Cell	Score	Instance ID
lisuride	12 µM	PC3	-1	6682
diphenylpyraline	13 µM	MCF7	-0.997	6061
oxamniquine	14 µM	PC3	-0.932	4006
paromomycin	6 µM	PC3	-0.865	4595
sirolimus	100 nM	MCF7	-0.852	5567
tretinoin	13 µM	MCF7	-0.84	3165
cyclobenzaprine	13 µM	PC3	-0.834	4252
metacycline	8 µM	HL60	-0.824	2901
valproic acid	50 µM	MCF7	-0.816	1002
cinnarizine	11 µM	HL60	-0.815	1558
oleandomycin	5 µM	PC3	-0.812	1935
cinnarizine	11 µM	PC3	-0.808	5817
CP-690334-01	1 µM	MCF7	-0.807	4383
PHA-00851261E	10 µM	PC3	-0.804	3773
iodixanol	3 µM	HL60	-0.799	3023
PHA-00745360	10 µM	PC3	-0.799	3824
primaquine	9 µM	PC3	-0.795	4263
articaine	12 µM	HL60	-0.795	3138
biperiden	11 µM	PC3	-0.793	4684
heliotrine	13 µM	PC3	-0.792	3717
betonicine	25 µM	PC3	-0.792	3745
diclofenamide	13 µM	PC3	-0.79	6686
clofazimine	8 µM	MCF7	-0.785	5642
(-)-isoprenaline	16 µM	MCF7	-0.783	6833
fluphenazine	10 µM	MCF7	-0.777	1017
rofecoxib	10 µM	MCF7	-0.775	166
myosmine	27 µM	MCF7	-0.773	6055

pivmecillinam	8 µM	MCF7	-0.773	6014
valproic acid	200 µM	MCF7	-0.773	994
azaperone	12 µM	PC3	-0.773	5877
thioperamide	10 µM	PC3	-0.772	4675
quercetin	12 µM	PC3	-0.769	4264
amprolium	13 µM	PC3	-0.766	4241
famotidine	12 µM	MCF7	-0.765	1529
betamethasone	10 µM	PC3	-0.765	6728
nicardipine	8 µM	MCF7	-0.765	5397
aminoglutethimide	17 µM	MCF7	-0.763	7421
isradipine	11 µM	PC3	-0.762	6347
CP-690334-01	1 µM	PC3	-0.761	4561
loxapine	9 µM	PC3	-0.759	6694
amrinone	21 µM	HL60	-0.759	2724
clemastine	9 µM	MCF7	-0.756	7485
saquinavir	5 µM	MCF7	-0.754	3549
nimodipine	10 µM	PC3	-0.754	6320
etilefrine	18 µM	MCF7	-0.753	7350
withaferin A	1 µM	MCF7	-0.752	3902
bendroflumethiazide	9 µM	PC3	-0.752	4315

HL60, human promyelocytic leukemia cell; PC3, human prostate cancer cell; MCF7, human breast cancer cell.

1.1 Supplementary Figures

Supplementary Figure. 1. Construction of the PPI network of DEGs. (**A-B**) PPI networks of DEGs in ccRCC (**A**) and pRCC (**B**) were constructed. (**C-D**) Networks indicate the hub genes for ccRCC (**C**) and pRCC (**D**) via the Cytoscape software.

Supplementary Figure. 2. GSEA enrichment analyses for *IGLL5* and/or *IL2RA* in ccRCC (A-B) and pRCC (C).

Supplementary Figure. 3. the correlation of *IGLL5* expression to immune cell abundance in ccRCC.

Supplementary Figure. 4. the correlation of *IL2RA* expression to immune cell abundance in ccRCC.

Supplementary Figure. 5. the correlation of *IL2RA* expression to immune cell abundance in pRCC.

Supplementary Figure. 6. The molecular structure of top 10 potential drugs for ccRCC (A-J) and pRCC (K-T).
